# Sex differences in childhood cancer risk among children with major birth defects: a Nordic population-based nested case-control study

**DOI:** 10.1093/ije/dyac192

**Published:** 2022-09-30

**Authors:** Dagrun Slettebø Daltveit, Kari Klungsøyr, Anders Engeland, Anders Ekbom, Mika Gissler, Ingrid Glimelius, Tom Grotmol, Laura Madanat-Harjuoja, Anne Gulbech Ording, Henrik Toft Sørensen, Rebecca Troisi, Tone Bjørge

**Affiliations:** Department of Global Public Health and Primary Care, University of Bergen, Bergen, Norway; Norwegian Quality Registry of Cleft Lip and Palate, Surgical Clinic, Haukeland University Hospital, Bergen, Norway; Department of Global Public Health and Primary Care, University of Bergen, Bergen, Norway; Division of Mental and Physical Health, Norwegian Institute of Public Health, Bergen, Norway; Department of Global Public Health and Primary Care, University of Bergen, Bergen, Norway; Division of Mental and Physical Health, Norwegian Institute of Public Health, Bergen, Norway; Clinical Epidemiology Division, Department of Medicine Solna, Karolinska Institutet, Stockholm, Sweden; Department of Knowledge Brokers, Finnish Institute for Health and Welfare, Helsinki, Finland; Region Stockholm, Academic Primary Health Care Centre, Stockholm, Sweden; Department of Molecular Medicine and Surgery, Karolinska Institutet, Stockholm, Sweden; Clinical Epidemiology Division, Department of Medicine Solna, Karolinska Institutet, Stockholm, Sweden; Department of Immunology, Genetics and Pathology, Uppsala University, Uppsala, Sweden; Cancer Registry of Norway, Oslo, Norway; Cancer Society of Finland, Finnish Cancer Registry, Helsinki, Finland; Dana Farber Cancer Institute, Boston Children’s Cancer and Blood Disorders Centre, Boston, MA, USA; Department of Clinical Epidemiology, Aarhus University Hospital and Aarhus University, Aarhus, Denmark; Department of Clinical Epidemiology, Aarhus University Hospital and Aarhus University, Aarhus, Denmark; Trans-divisional Research Program, Division of Cancer Epidemiology and Genetics, National Cancer Institute, Rockville, MD, USA; Department of Global Public Health and Primary Care, University of Bergen, Bergen, Norway; Cancer Registry of Norway, Oslo, Norway

**Keywords:** Childhood cancer, birth defects, congenital anomalies, sex differences, cancer risk

## Abstract

**Background:**

Childhood cancer is more common among children with birth defects, suggesting a common aetiology. Whether this association differs by sex is unclear.

**Methods:**

We performed a population-based nested case-control study using nationwide health registries in four Nordic countries. We included 21 898 cancer cases (0–19 years) and 218 980 matched population controls, born 1967–2014. Associations between childhood cancer and major birth defects were calculated as odds ratios (ORs) with 95% confidence intervals (CIs) using logistic regression models. Effect modification was evaluated using a counterfactual framework to estimate confidence intervals and *P*-values for the natural indirect effects.

**Results:**

Birth defects were present for 5.1% (1117/21 898) of childhood cancer cases and 2.2% (4873/218 980) of controls; OR of cancer was higher for chromosomal (OR = 10, 95% CI = 8.6–12) than for non-chromosomal defects (OR = 1.9, 95% CI = 1.8–2.1), strongest between genetic syndromes/microdeletion and renal tumours, Down syndrome and leukaemia, and nervous system defects and central nervous system tumours. The association between birth defects and cancer was stronger among females (OR = 2.8, 95% CI = 2.6–3.1) than males (OR = 2.1, 95% CI = 1.9–2.2, *P*_interaction_ <0.001). Male sex was an independent risk factor for childhood cancer, but very little of the overall association between sex and childhood cancer was mediated through birth defects (4.8%, *P*_NIE_ <0.001), although more at younger ages (10% below years and 28% below 1 year).

**Conclusions:**

The birth defect–cancer associations were generally stronger among females than males. Birth defects did not act as a strong mediator for the modest differences in childhood cancer risk by sex, suggesting that other biological pathways are involved.

Key Messages

- Having a birth defect is one of the strongest confirmed risk factors for childhood cancer.
- In this large population-based nested case-control study of more than 21 000 incident childhood cancer cases, we observed sex differences in the birth defect–cancer associations.
- Our study indicates that the birth defect–cancer associations, in general, are stronger among females than males, particularly for non-chromosomal defects and lymphomas and germ cell tumours, and chromosomal defects and leukaemia.
- We did not find evidence supporting the hypothesized role of birth defects as a strong mediator in the sex–childhood cancer association.
- The sex differences in the birth defect–cancer association suggest that further studies on the underlying mechanisms are needed.

## Introduction

Globally, approximately 400 000 new childhood cancer cases (ages 0–19 years) are diagnosed each year, and the estimated age-standardized incidence rate is 16.2 per 100 000 person-years.^1^ The global burden of childhood cancer is unequally distributed, with 82% of disability-adjusted life-years (DALYs) due to childhood cancer occurring in resource-limited populations (which include more than 90% of children at risk of cancer).^1^ Still, few strong risk factors for childhood cancer have been identified.^2^

Existing evidence of an association between birth defects and childhood cancer ^3–5^ suggests a common aetiology. Increases in childhood cancer risk are observed for both chromosomal (∼11-fold) and non-chromosomal (∼2–3-fold) birth defects.^3^^,^^4^ Associations between several specific birth defects and childhood cancers have been identified (e.g. Down syndrome and leukaemia, central nervous system (CNS) defects and CNS tumours), and a positive risk gradient by the number of birth defects has been observed.^3–5^ There is also evidence that the increased cancer risk among individuals with birth defects persists into adulthood.^3^

Approximately 2% to 3% of liveborn children in the Nordic countries have major birth defects.^6^ The prevalence of birth defects and incidence of childhood cancer are higher among males than females (∼1.2-fold).^7^^,^^8^ Like childhood cancer, most birth defects have an unknown aetiology.^9^ Although the association between birth defects and childhood cancer is well established, research on possible sex differences in this association is sparse.^10–12^ However, a recent study suggests that birth defects may act as a strong mediator, explaining up to 40% of the association between sex and childhood cancer.^13^

Large populations are needed to study associations between birth defects and childhood cancer, particularly by sex, since the frequencies of both conditions are low. By linking national registries in four Nordic countries, we examined the risk of cancer before the age of 20 years among individuals with birth defects by sex and evaluated the role of birth defects as a mediator in the sex–childhood cancer relationship.

## Methods

### Data sources and study population

The Nordic countries have high-quality national population-based health registries with close to complete nationwide coverage, and unique personal identification numbers for all individuals residing in the countries facilitate accurate linkage between registries.^3^^,^^14^ We performed a nested case-control study and defined cases as individuals recorded in the medical birth registries with diagnoses in the cancer registries before the age of 20 years (born from 1977 in Denmark, 1987 in Finland, 1967 in Norway and 1973 in Sweden). Controls were frequency-matched on country and year of birth among persons who were alive, residing in the country and with no cancer diagnoses by the end of follow-up (2013 in Denmark, Finland, and Norway; 2014 in Sweden).

We obtained information on cancer diagnoses from cancer registries. The cancer registries have close to complete coverage of the entire populations from 1943 in Denmark, 1953 in Finland and Norway and 1958 in Sweden, with minor variations in completeness between countries, time periods and age at diagnosis.^15–20^ Information on birth defects was collected from the medical birth registries, congenital malformations registry (Finland) and patient registries (Denmark and Sweden).^21–23^ From the patient registries, we included birth defects identified during hospitalizations in the first year of life. Information on death and emigration was obtained from the national population registries. The data sources have been described in detail previously^3^ (see Supplementary Table S1, available as Supplementary data at *IJE* online, for more details).

### Birth defect classification

Major birth defects were defined and classified according to the European network of population-based registries for the epidemiological surveillance of congenital anomalies (EUROCAT).^24^ Isolated birth defects were defined as single birth defects, multiple defects within the same anatomical subgroup, or multiple defects when part of a sequence. Multiple birth defects were defined as birth defects from different anatomical subgroups that are not part of a sequence.^3^

### Childhood cancer classification

Cancer cases were classified according to the *International Classification of Childhood Cancer,* Third Edition (ICCC-3) (IARC 2017).^25^ We excluded non-malignant neoplasms, except for central nervous system (CNS) tumours (ICCC-3 site group III, CNS and Miscellaneous Intracranial and Intraspinal Neoplasms) and intracranial and intraspinal germ cell tumours [ICCC-3 site group X(a)], cases without verified morphology and cases not classified by the ICCC-3.

### Statistical analysis

We used unconditional logistic regression to compute odds ratios (ORs) of cancer with 95% confidence intervals (CIs), comparing individuals with and without major birth defects. ORs were adjusted for sex and matching factors. Other available confounders considered were in vitro fertilization (IVF), maternal age and smoking. Information on IVF (not available for Denmark) and smoking was not available for the entire study period. Potential confounding was evaluated by comparing estimates with and without these factors included in the models, using a complete case approach for handling missing data. To evaluate the robustness of the results, we calculated E-values for the OR and the lower confidence limit.^26^ Analyses stratified by sex and analyses including a sex–birth defect interaction term were performed to evaluate possible sex differences in birth defect–cancer associations. Chromosomal and non-chromosomal birth defects were analysed separately.

To evaluate birth defects as a potential mediator of the sex–childhood cancer association, we used a counterfactual framework allowing for exposure–mediator interaction. We estimated the controlled direct effect (CDE), the natural direct effect (NDE), the natural indirect effect (NIE) and the marginal total effect (TE, i.e. the product of NDE and NIE).^27^ We included a sex–birth defect interaction and adjusted for the following potential mediator–outcome confounders: birth year, country and maternal age. Also, we performed sensitivity analyses where we included IVF and maternal smoking as confounders. To assess whether effect modification was present, we used CIs and *P*-values for the NIE and calculated the proportion of the sex effect mediated through birth defects.^27^Supplementary Figure S1 (available as Supplementary data at *IJE* online) shows a simplified illustration of the assumed causal relationship between sex, birth defects and childhood cancer.

Given differences in registries and time periods, we performed additional sensitivity analyses leaving out one country at a time. Also, to evaluate the possible impact of diagnostic and survival trends on our results, we performed sensitivity analyses limited to the ∼60% of the cases and controls born in 1990 and after.

All analyses were performed using Stata version 16, and causal mediation effects were estimated using the Stata PARAMED macro.

## Results

In all, 21 898 children were diagnosed with cancer during the study period. The largest malignancy group was leukaemia (*n* = 5552, 25%), followed by CNS tumours (*n *= 5177, 24%) and lymphomas (*n* = 2907, 13%). Among cancer cases, 5.1% (*n* = 1117) were born with major birth defects, compared with 2.2% (*n *= 4873) among controls. The three largest birth defect categories were congenital heart defects (*n* = 1754, 0.73%), limb defects (*n *= 1017, 0.42%) and genital defects (*n* = 600, 0.25%). Median age at primary cancer diagnosis was 8 years (interquartile range: 3 to 15 years), and 38% (8259/21 898) were diagnosed with cancer before the age of 5 years (Table 1). The overall male-to-female ratio of cancer was 1.14, and the male-to-female ratio of any birth defect was 1.30 (Supplementary Tables S2 and S3, respectively, available as Supplementary data at *IJE* online).

**Table 1:** Characteristics of the study population in Denmark (1977–2013), Finland (1987–2013), Norway (1967–2013) and Sweden (1973–2014)

Characteristics	Cases		Controls	
	*n*	%	*n*	%
Study population	21 898	9.1	218 980	90.9
Major birth defects	1117	5.1	4873	2.2
Sex^a^				
Males	11 937	54.5	111 260	50.8
Females	9961	45.5	107 720	49.2
Birthweight (g)				
< 2500	942	4.3	9104	4.2
2500–3999	16 301	74.4	169 802	77.5
4000 or more	4573	20.9	39 403	18.0
Missing	82	0.4	671	0.3
Gestational age (weeks)				
< 37	1336	6.1	11 730	5.4
37–41	18 172	83.0	183 176	83.6
42 or more	1832	8.4	18 541	8.5
Missing	558	2.5	5533	2.5
In vitro fertilization^b^				
No	7778	55.7	78 003	55.9
Yes	127	0.9	1047	0.7
Not collected	6056	43.4	60 560	43.4
Maternal smoking^c^				
No	10 612	48.5	105 339	48.1
Yes	2391	10.9	24 872	11.4
Missing^d^	958	6.9	9399	6.7
Not collected	8895	40.6	88 769	40.5
Maternal age (years)				
< 25	5164	23.6	58 481	26.7
25–29	7744	35.4	79 584	36.3
30–34	6029	27.5	56 009	25.6
35 or more	2961	13.5	24 906	11.4
Paternal age (years)^e^				
< 25	1257	5.7	13 015	5.9
25–29	2666	12.2	26 599	12.1
30–34	2562	11.7	25 886	11.8
35 or more	2161	9.9	20 835	9.5
Missing	13 252	60.5	132 645	60.6
Year of birth				
1967–1970	525	2.4	5250	2.4
1970–1979	2541	11.6	25 410	11.6
1980–1989	5405	24.7	54 050	24.7
1990–1999	8285	37.8	82 850	37.8
2000–2009	4418	20.2	44 180	20.2
2010–2014	724	3.3	7240	3.3
Age at primary cancer diagnosis (years)^f^				
0–4	8259	37.7		
5–9	4109	18.8		
10–14	3774	17.2		
15–19	5756	26.3		
Year of primary cancer diagnosis^f^				
Before 1980	798	3.6		
1980–1989	1961	9.0		
1990–1999	6146	28.1		
2000–2009	8572	39.1		
2010 or later	4421	20.2		

a Differences between cases and controls were due to birth:sex ratio and different cancer risk for males and females in the study population.

b Reported from 1990 in Finland, 1984 in Norway and 1995 in Sweden; not included for Denmark.

c Available from 1991 in Denmark, 1987 in Finland, 1998 in Norway and 1982 in Sweden.

d Percentage missing in the time period when this information was recorded.

e Not reported in Sweden and Finland.

f Reported only for cases.

### Risk of any and specific cancers

**Figure 1:**
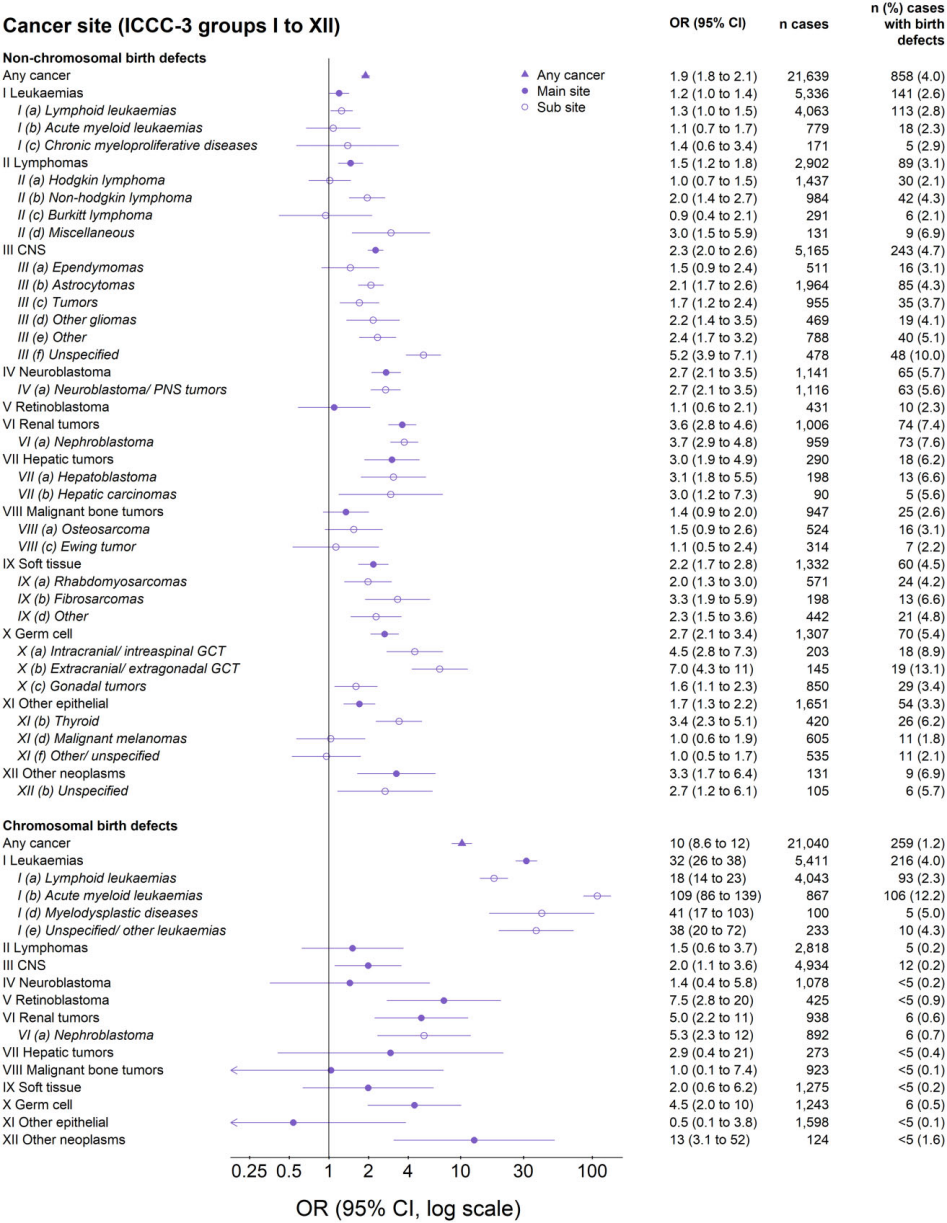
Risk of specific cancers in individuals with any major birth defect. Odds ratios (ORs) adjusted for matching variables (birth year and country) and sex. Adding additional confounders during the period when these were recorded did not change the results and was not included in the final models. Cancers classified into International Classification of Childhood Cancer, Third Edition (ICCC-3) groups I-XII (not included are sites with less than five co-occurring birth defects and cancers). OR, odds ratio; CI, confidence interval; CNS, central nervous system; PNS, peripheral nervous system; GCT, germ cell tumour

The OR of cancer for children with major birth defects was higher for chromosomal (OR 10, 95% CI 8.6–12) than for non-chromosomal defects (1.9, 1.8–2.1; Figure 1). ORs were adjusted for country, birth year and sex. Additional adjustment for IVF, maternal age and smoking, during the time period when these were recorded, did not change the results and were not included in the final models (Supplementary Tables S4–S6, available as Supplementary data at *IJE* online). The highest risk was observed among children with Down syndrome (12, 9.9–14), followed by genetic syndromes/microdeletions (7.0, 4.1–12) and nervous system defects (6.1, 4.7–7.9; Figure 2). Also, children with skeletal dysplasia and defects of the eye, digestive system, urinary system, limbs, heart and other defects had an increased overall cancer risk. The strongest associations between specific birth defects and specific cancers were observed for genetic syndromes/microdeletion and renal tumours (55; 26–117), Down syndrome and leukaemia (41, 33–49), and nervous system defects and central nervous system tumours (16, 12–22). Cancer risks increased by number of birth defects and were greatest for the youngest children (Supplementary Figures S2 and S3, available as Supplementary data at *IJE* online). Specifically among children with Down syndrome, the risk of acute lymphoid leukaemia (ALL) increased by age at diagnosis: OR = 12, 22 and 27 for ages <2, 2–4 and ≥5 years, respectively. Also, the risk of acute myeloid leukaemia (AML) was extremely high before the age of five, with few cases with Down syndrome above the age of five: OR = 253, 451, 256 and 7.7 for ages <1, 1, 2–4, and ≥5 years, respectively (Supplementary Table S7, available as Supplementary data at *IJE* online).

**Figure 2:**
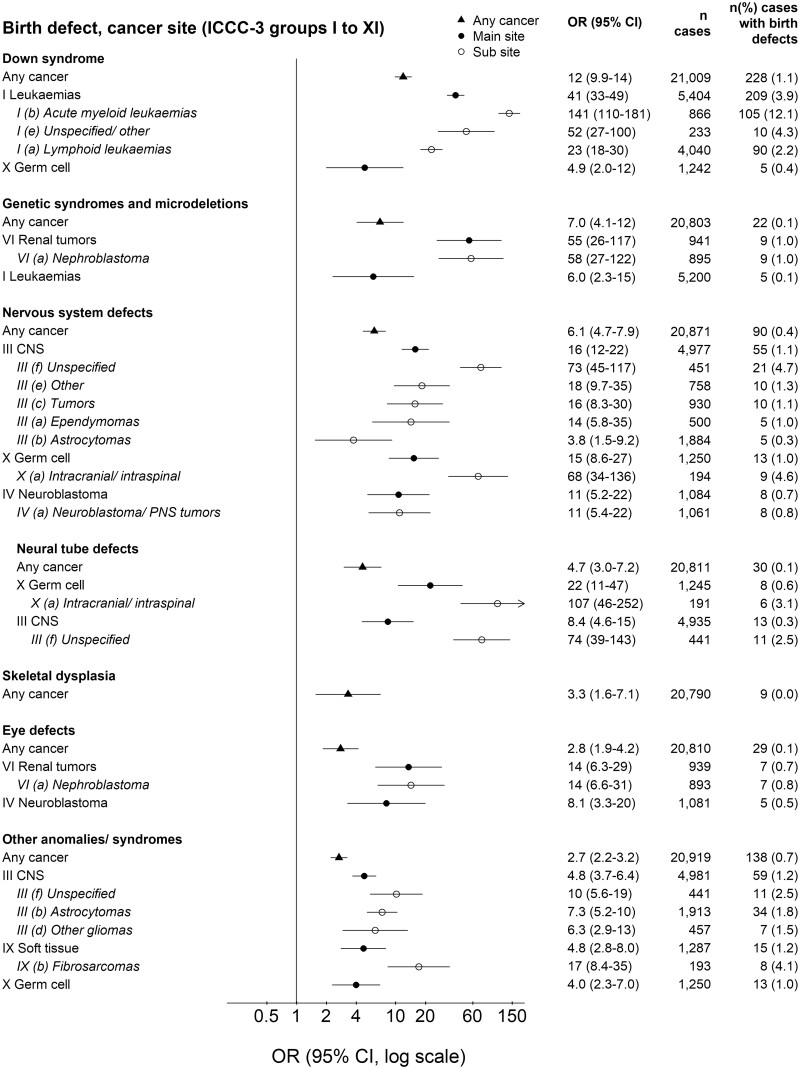
Associations between specific major birth defects and any or specific cancers. Odds ratios (ORs) adjusted for matching variables (birth year and country) and sex. Cancers classified into International Classification of Childhood Cancer, Third Edition (ICCC-3) groups I-XII (not included are sites with less than five co-occurring birth defects and cancers). Other anomalies/syndromes include, among others, congenital skin disorders (*n* = 158), craniosynostosis (*n* = 55), neurofibromatosis (*n* = 52), tuberous sclerosis (*n* = 37), vascular disruption anomalies (*n* = 36) and teratogenic syndromes with malformations (*n* = 30). Analyses of specific non-chromosomal birth defects included only isolated defects, see Supplementary Table S12 (available as Supplementary data at *IJE* online) for additional combinations of birth defects and childhood cancer. OR, odds ratio; CI, confidence interval; CNS, central nervous system; PNS, peripheral nervous system

**Figure 2:**
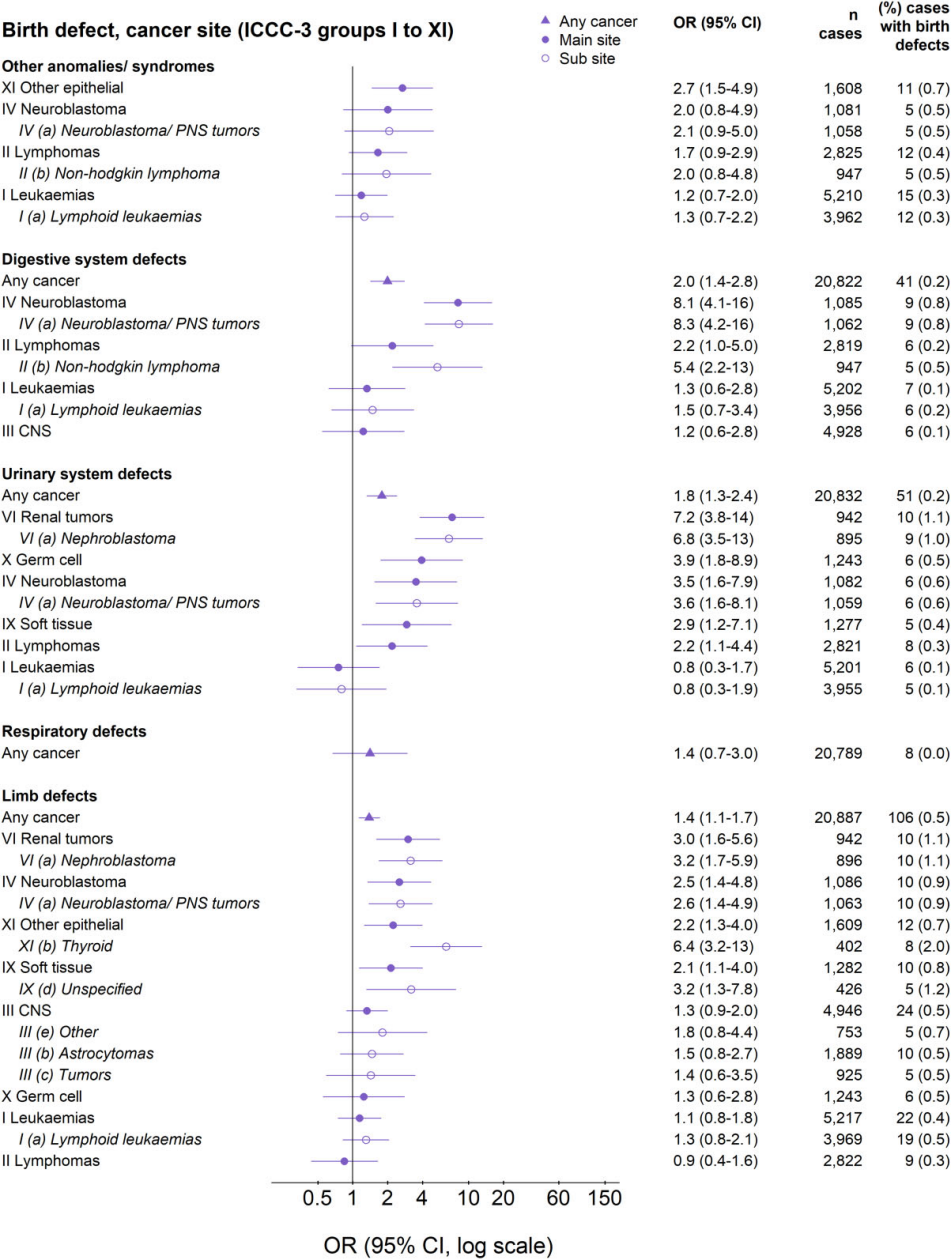
(Continued)

**Figure 2:**
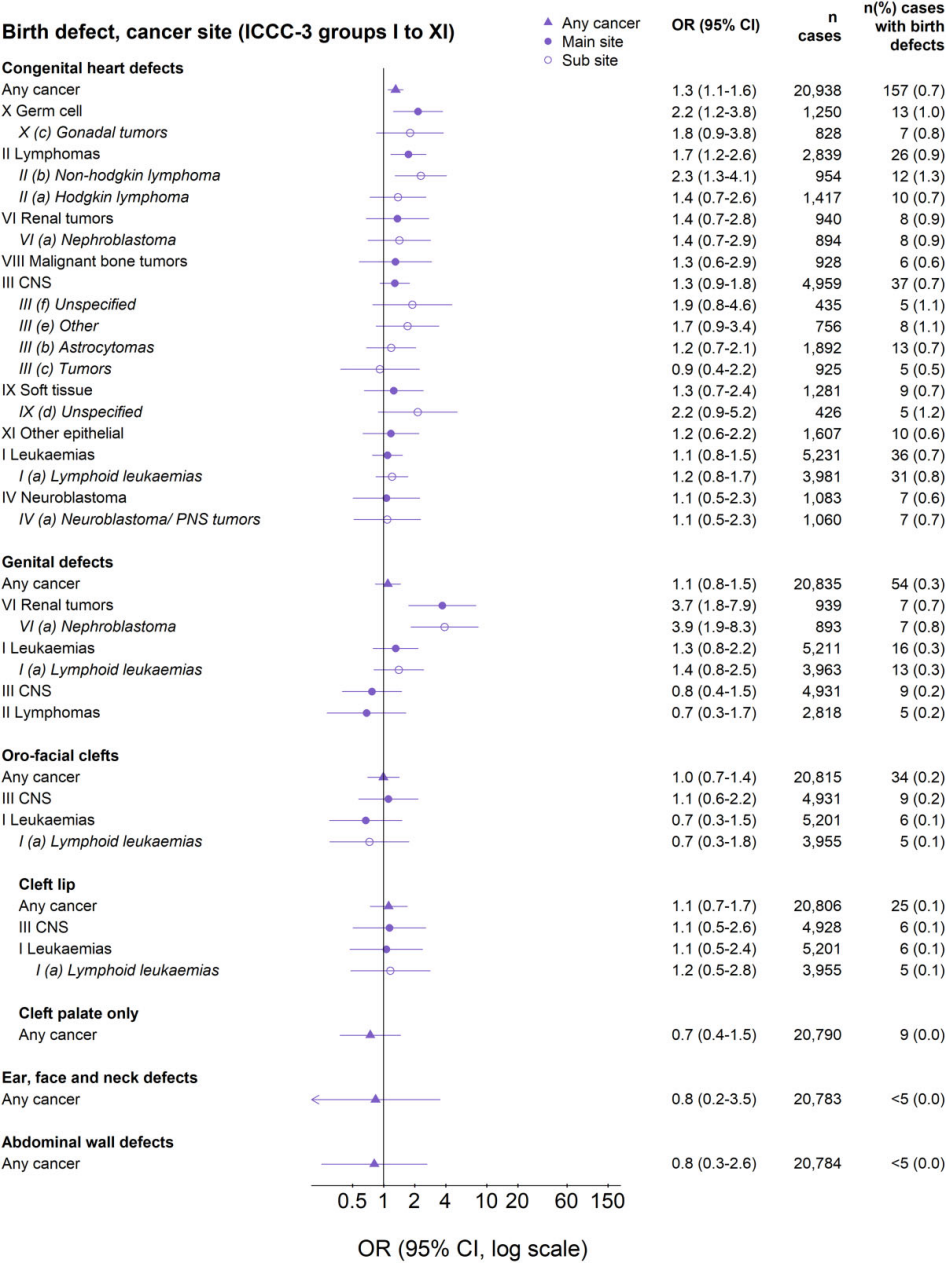
(Continued)

### Sex differences in the association between birth defects and cancer

The association between birth defects and risk of any cancer differed for males and females (Table 2). The OR of cancer among males with any birth defect was 2.1 (1.9–2.3) compared with 2.8 (2.6–3.1) among females (*P*_interaction_ <0.001). Results were similar when chromosomal defects were excluded [males: 1.7 (1.6–1.9) and females: 2.2 (2.0–2.5), *P*_interaction_ = 0.001]. When examining specific birth defects in relation to any cancer, the effect sizes were mostly larger in females than males, for instance for urinary system defects [males: 1.3 (0.9–2.0) and females: 2.8 (1.8–4.5), *P*_interaction_ = 0.053] and genital organs defects [males: 1.0 (0.8–1.4) and females: 2.4 (1.8–5.0), *P*_interaction_ = 0.052]. Also, when investigating associations between any birth defect and specific cancers, we observed sex differences (Table 3). The effect sizes were greater among females than males for the majority of cancer sites, and interactions were observed for non-chromosomal birth defects and lymphomas [males: 1.2 (0.9–1.6) and females: 2.0 (1.4–2.7), *P*_interaction_ = 0.04], non-chromosomal birth defects and germ cell tumours [males: 2.0 (1.4–2.7) and females: 4.8 (3.3–6.9), *P*_interaction_ <0.001] and chromosomal birth defects and leukaemia [males: 26 (20–33) and females: 39 (30–50), *P*_interaction_ = 0.02]. The female birth–defect cancer associations were stronger than among males at all ages (Supplementary Table S8, available as Supplementary data at *IJE* online).

**Table 2:** Risk of any cancer among children with birth defects, stratified by sex

	Males			Females			*P*_interaction_
Birth defects	Cases *n* (%)	Controls *n* (%)	OR (95% CI)	Cases *n* (%)	Controls *n* (%)	OR (95% CI)	
All anomalies	608 (5.1%)	2848 (2.6%)	2.1 (1.9–2.2)	509 (5.1%)	2025 (1.9%)	2.8 (2.6–3.1)	<0.001
All anomalies excluding chromosomal anomalies	486 (4.1%)	2716 (2.4%)	1.7 (1.6–1.9)	372 (3.8%)	1893 (1.8%)	2.2 (2.0–2.5)	0.001
**Specific sites**							
Nervous system	48 (0.4%)	70 (0.1%)	6.6 (4.5–9.5)	42 (0.4%)	83 (0.1%)	5.7 (3.9–8.2)	0.59
Neural tube defects	15 (0.1%)	28 (0%)	5.1 (2.7–9.6)	15 (0.2%)	39 (0%)	4.3 (2.4–7.8)	0.70
Eye	12 (0.1%)	57 (0.1%)	2.0 (1.1–3.7)	17 (0.2%)	51 (0%)	3.8 (2.2–6.6)	0.15
Ear, face and neck	<5 (0%)	14 (0%)	NA	<5 (0%)	11 (0%)	2.1 (0.5–9.6)	NA
Congenital heart defects	79 (0.7%)	599 (0.5%)	1.3 (1.0–1.6)	78 (0.8%)	650 (0.6%)	1.3 (1.1–1.7)	0.71
Respiratory system	<5 (0%)	25 (0%)	1.2 (0.3–3.8)	5 (0.1%)	34 (0%)	1.6 (0.6–4.2)	0.65
Orofacial clefts	24 (0.2%)	203 (0.2%)	1.1 (0.7–1.7)	10 (0.1%)	144 (0.1%)	0.8 (0.4–1.5)	0.34
Cleft palate only	<5 (0%)	53 (0%)	0.5 (0.2–1.7)	6 (0.1%)	75 (0.1%)	0.9 (0.4–2.1)	0.49
Cleft lip with/without cleft palate	21 (0.2%)	153 (0.1%)	1.3 (0.8–2.1)	<5 (0%)	69 (0.1%)	0.6 (0.2–1.8)	0.21
Digestive system	22 (0.2%)	106 (0.1%)	2.0 (1.3–3.2)	19 (0.2%)	106 (0.1%)	2.0 (1.2–3.3)	0.97
Abdominal wall defects	<5 (0%)	20 (0%)	1.0 (0.2–4.1)	<5 (0%)	18 (0%)	0.6 (0.1–4.7)	0.73
Urinary system	29 (0.3%)	195 (0.2%)	1.4 (1.0–2.1)	22 (0.2%)	95 (0.1%)	2.6 (1.6–4.2)	0.05
Genital organs	46 (0.4%)	434 (0.4%)	1.0 (0.8–1.4)	8 (0.1%)	40 (0%)	2.4 (1.1–5.0)	0.05
Limb	61 (0.5%)	482 (0.4%)	1.2 (0.9–1.6)	45 (0.5%)	290 (0.3%)	1.7 (1.3–2.4)	0.07
Skeletal dysplasia	<5 (0%)	17 (0%)	2.2 (0.8–6.7)	5 (0.1%)	11 (0%)	5.3 (1.8–15)	0.29
Genetic syndromes and microdeletions	12 (0.1%)	18 (0%)	6.4 (3.1–13)	10 (0.1%)	15 (0%)	7.5 (3.4–17)	0.79
Chromosomal	122 (1.1%)	132 (0.1%)	9.0 (7.0–12)	137 (1.4%)	132 (0.1%)	12 (9.1–15)	0.13
Down syndrome	107 (0.9%)	98 (0.1%)	11 (8.1–14)	121 (1.3%)	101 (0.1%)	13 (10.3–17)	0.21
Other anomalies/syndromes	73 (0.6%)	306 (0.3%)	2.3 (1.8–2.9)	65 (0.7%)	220 (0.2%)	3.3 (2.5–4.4)	0.06

OR, odds ratio; CI, confidence interval.

**Table 3:** Risk of specific cancers in individuals with any major birth defect, stratified by sex

Cancer site	Males			Females			*P*_interaction_
	No. cases	No. (%) cases with BD	OR (95% CI)	No. cases	No. (%) cases with BD	OR (95% CI)	
**Non-chromosomal birth defects**							
I Leukaemias, myeloproliferative and myelodysplastic diseases	2942	87 (3.0%)	1.2 (1.0–1.5)	2394	54 (2.3%)	1.2 (0.9–1.6)	0.86
II Lymphomas and reticuloendothelial neoplasms	1765	52 (2.9%)	1.2 (0.9–1.6)	1137	37 (3.3%)	2.0 (1.4–2.7)	0.04
III CNS and miscellaneous intracranial and intraspinal neoplasms	2790	137 (4.9%)	2.1 (1.7–2.4)	2375	106 (4.5%)	2.6 (2.2–3.2)	0.08
IV Neuroblastoma and other peripheral nervous cell tumours	623	36 (5.8%)	2.4 (1.7–3.4)	518	29 (5.6%)	3.2 (2.2–4.7)	0.28
V Retinoblastoma	231	5 (2.2%)	0.9 (0.4–2.2)	200	5 (2.5%)	1.4 (0.6–3.5)	0.46
VI Renal tumours	484	36 (7.4%)	3.1 (2.2–4.4)	522	38 (7.3%)	4.2 (3.0–5.9)	0.23
VII Hepatic tumours	173	10 (5.8%)	2.5 (1.3–4.7)	117	8 (6.8%)	4.1 (2.0–8.4)	0.30
VIII Malignant bone tumours	518	16 (3.1%)	1.3 (0.8–2.2)	429	9 (2.1%)	1.4 (0.7–2.6)	0.98
IX Soft-tissue and other extraosseous sarcomas	747	40 (5.4%)	2.3 (1.6–3.1)	585	20 (3.4%)	2,0 (1.3–3.2)	0.67
IX (a) Rhabdomyosarcoma	330	15 (4.5%)	1.9 (1.1–3.2)	241	9 (3.7%)	2.1 (1.1–4.2)	0.76
X Germ cell tumours, trophoblastic tumours and neoplasms of gonads	908	40 (4.4%)	2.0 (1.4–2.7)	399	30 (7.5%)	4.8 (3.3–6.9)	<0.001
XI Other malignant epithelial neoplasms and malignant melanomas	580	23 (4.0%)	1.7 (1.1–2.5)	1071	31 (2.9%)	1.8 (1.2–2.5)	0.87
XII Other and unspecified malignant neoplasms	54	<5 (7.4%)	3.1 (1.1–8.7)	77	5 (6.5%)	3.4 (1.4–8.4)	0.81
**Chromosomal birth defects**							
I Leukaemias, myeloproliferative and myelodysplastic diseases	2951	96 (3.3%)	26 (20–33)	2460	120 (4.9%)	39 (30–50)	0.02
III CNS and miscellaneous intracranial and intraspinal neoplasms	2661	8 (0.3%)	2.5 (1.2–5.0)	2273	<5 (0.2%)	1.4 (0.5–3.9)	0.36
VI Renal tumours (a.1 nephroblastoma)	419	<5 (0.2%)	1.9 (0.3–14)	456	5 (1.1%)	8.7 (3.5–21)	0.16
X Germ cell tumours, trophoblastic tumours and neoplasms of gonads	874	6 (0.7%)	6.8 (3.0–16)	369	<5 (0%)	NA	NA

ORs are adjusted for matching variables (birth-year and country). Not included are cancers classified in ICCC-3 groups and subsites with less than five co-occurring birth defects and cancers (for both males and females).

BD, birth defect; CNS, central nervous system.

### Birth defects as a mediator for the association between sex and childhood cancer

Analysing sex separately as a risk factor for childhood cancer resulted in a male-to-female OR for any cancer of 1.16 (1.13–1.19), adjusted for birth year and country (Supplementary Table S2). Males had an increased risk of cancer for most cancer sites, lymphomas and germ cell tumours in particular, whereas females had an increased risk of other malignant epithelial neoplasms and malignant melanomas. Birth defects appeared to mediate very little of the overall association between sex and childhood cancer risk (proportion mediated: 4.8%, *P*_NIE_ <0.001; Table 4). Specifically, we observed evidence of mediation for the risk of neuroblastoma and other peripheral nervous system tumours (6.5%, *P*_NIE_ = 0.001), leukaemia (6.0%, *P*_NIE_ <0.001), CNS tumours (5.7%, *P*_NIE_ <0.001), soft-tissue sarcomas (4.2%, *P*_NIE_ = 0.001), and germ cell tumours (1.3%, *P*_NIE_ = 0.001). Among children diagnosed with cancer before the age of five, the proportion mediated by birth defects was larger (11%, *P*_NIE_ <0.001). Mediation was observed for CNS tumours (8.2%, *P*_NIE_ <0.001) and soft-tissue sarcomas (4.1%, *P*_NIE_ = 0.001). For children diagnosed with cancer before the age of one, 28% (*P*_NIE_ <0.001) of the male sex effect was mediated by birth defects. Separate analyses excluding chromosomal birth defects resulted in lower percentages mediated for overall cancer among children of all ages (Supplementary Table S9, available as Supplementary data at *IJE* online). Sensitivity analyses where we adjusted for potential mediator–outcome confounders (IVF and smoking) did not alter the results (Supplementary Table S10, available as Supplementary data at *IJE* online).

**Table 4:** Mediation analyses of the effect of birth defects on the association between sex (males versus females) and childhood cancer, overall and by cancer site^a^

Cancer site	Controlled direct effect^b^ (CDE) OR (95% CI)	Natural indirect effect^c^ (NIE) OR (95% CI)	Natural direct effect^d^ (NDE) OR (95% CI)	Marginal total effect (MTE) OR (95% CI)	Percentage (%) mediated^e^
Total study population (0–19 years)					
Any cancer	1.17 (1.14–1.20)	1.007 (1.005–1.008)	1.16 (1.12–1.19)	1.16 (1.13–1.20)	4.80
I Leukaemias, myeloproliferative and myelodysplastic diseases	1.19 (1.12–1.25)	1.009 (1.006–1.012)	1.16 (1.10–1.22)	1.17 (1.11–1.24)	5.95
(a) Lymphoid leukaemias	1.19 (1.12–1.27)	1.005 (1.003–1.008)	1.18 (1.11–1.25)	1.18 (1.11–1.26)	3.42
Other leukaemias	1.33 (1.11–1.60)	1.008 (1.001–1.015)	1.32 (1.11–1.57)	1.33 (1.11–1.58)	3.16
II Lymphomas and reticuloendothelial neoplasms	1.53 (1.42–1.66)	1.002 (1.000–1.004)	1.52 (1.41–1.63)	1.52 (1.41–1.64)	NA
(b) Non-Hodgkin lymphomas	1.95 (1.71–2.24)	1.004 (1.000–1.009)	1.92 (1.68–2.19)	1.93 (1.69–2.20)	0.92
Other lymphomas	2.92 (2.37–3.61)	1.003 (0.998–1.009)	2.88 (2.34–3.54)	2.89 (2.35–3.55)	NA
III CNS and miscellaneous intracranial and intraspinal neoplasms	1.14 (1.08–1.21)	1.007 (1.004–1.010)	1.13 (1.08–1.21)	1.14 (1.08–1.20)	5.69
(a.1) Ependymomas	1.30 (1.06–1.59)	1.000 (0.995–1.006)	1.29 (1.06–1.58)	1.29 (1.06–1.58)	NA
(c.1) Medulloblastomas	1.70 (1.44–2.00)	1.000 (0.996–1.004)	1.64 (1.40–1.93)	1.64 (1.40–1.93)	NA
(c.2) Primitive neuroectodermal tumour	1.36 (1.06–1.74)	1.003 (0.995–1.011)	1.31 (1.03–1.68)	1.32 (1.03–1.68)	NA
IV Neuroblastoma and other peripheral nervous cell tumours	1.16 (1.03–1.31)	1.009 (1.004–1.014)	1.15 (1.02–1.29)	1.16 (1.03–1.30)	6.52
(a) Neuroblastoma	1.14 (1.01–1.29)	1.009 (1.004–1.014)	1.13 (1.01–1.27)	1.14 (1.01–1.29)	7.08
VII Hepatic tumours	1.44 (1.13–1.84)	1.009 (0.999–1.018)	1.40 (1.11–1.77)	1.41 (1.12–1.78)	2.94
(a.1) Hepatoblastoma	1.69 (1.25–2.29)	1.006 (0.996–1.017)	1.59 (1.19–2.13)	1.60 (1.19–2.14)	NA
VIII Malignant bone tumours	1.16 (1.02–1.32)	1.002 (0.999–1.006)	1.16 (1.02–1.32)	1.17 (1.03–1.33)	NA
(a) Osteosarcoma	1.25 (1.05–1.49)	1.002 (0.996–1.007)	1.23 (1.04–1.47)	1.24 (1.04–1.47)	NA
IX Soft-tissue and other extraosseous sarcomas	1.22 (1.09–1.36)	1.008 (1.003–1.013)	1.22 (1.10–1.37)	1.23 (1.11–1.38)	4.18
(a) Rhabdomyosarcoma	1.31 (1.11–1.56)	1.005 (0.999–1.011)	1.31 (1.11–1.55)	1.32 (1.12–1.56)	NA
Other soft tissue	1.16 (1.00–1.34)	1.010 (1.003–1.017)	1.16 (1.01–1.34)	1.18 (1.02–1.36)	6.68
X Germ cell tumours, trophoblastic tumours and neoplasms of gonads	2.30 (2.04–2.60)	1.007 (1.003–1.012)	2.21 (1.96–2.48)	2.22 (1.97–2.50)	1.33
(a) Intracranial germ cell tumours	2.09 (1.53–2.84)	1.015 (1.002–1.029)	1.95 (1.45–2.61)	1.98 (1.48–2.65)	3.08
(b) Extracranial germ cell tumours	0.68 (0.48–0.97)	1.025 (1.002–1.048)	0.64 (0.46–0.90)	0.66 (0.47–0.92)	NA
(c), (d), and (e) Gonadal germ cell tumours	2.86 (2.47–3.32)	1.004 (1.000–1.009)	2.83 (2.45–3.28)	2.85 (2.46–3.29)	0.67
XI Other malignant epithelial neoplasms and malignant melanomas	0.53 (0.48–0.59)	1.004 (1.000–1.009)	0.53 (0.48–0.59)	0.53 (0.48–0.59)	NA
XII Other and unspecified malignant neoplasms	0.68 (0.47–0.97)	1.016 (0.996–1.036)	0.67 (0.48–0.95)	0.68 (0.48–0.97)	NA
Children younger than 5 years at time of diagnosis					
Any cancer	1.13 (1.08–1.18)	1.011 (1.008–1.014)	1.10 (1.05–1.15)	1.11 (1.06–1.16)	10.69
II Lymphomas and reticuloendothelial neoplasms	2.28 (1.82–2.85)	1.006 (0.999–1.013)	2.27 (1.82–2.83)	2.28 (1.83–2.84)	NA
III CNS and miscellaneous intracranial and intraspinal neoplasms	1.16 (1.05–1.28)	1.013 (1.008–1.018)	1.16 (1.05–1.27)	1.17 (1.07–1.29)	8.21
VII Hepatic tumours	1.78 (1.31–2.41)	1.004 (0.995–1.013)	1.64 (1.22–2.19)	1.64 (1.23–2.20)	NA
IX Soft-tissue and other extraosseous sarcomas	1.29 (1.07–1.56)	1.009 (1.001–1.017)	1.27 (1.06–1.53)	1.28 (1.07–1.54)	4.14
X Germ cell tumours, trophoblastic tumours and neoplasms of gonads	1.58 (1.22–2.04)	1.007 (0.998–1.017)	1.38 (1.09–1.75)	1.39 (1.09–1.77)	NA
XII Other and unspecified malignant neoplasms	0.56 (0.35–0.91)	1.025 (0.995–1.057)	0.57 (0.36–0.89)	0.58 (0.37–0.91)	NA
Children younger than 1 year at time of diagnosis					
Any cancer	1.10 (0.99–1.21)	1.025 (1.018–1.033)	1.06 (0.97–1.17)	1.09 (0.99–1.20)	28.15
I Leukaemias, myeloproliferative and myelodysplastic diseases	0.67 (0.53–0.86)	1.043 (1.023–1.063)	0.70 (0.56–0.88)	0.73 (0.59–0.91)	NA
IV Neuroblastoma and other peripheral nervous cell tumours	1.40 (1.14–1.73)	1.020 (1.009–1.031)	1.36 (1.12–1.66)	1.39 (1.14–1.70)	6.85
XII Other and unspecified malignant neoplasms	0.45 (0.23-0.91)	1.044 (0.990-1.102)	0.47 (0.25-0.88)	0.49 (0.26-0.91)	NA

OR, odds ratio; CI, confidence interval; CNS, central nervous system.

a A causal interpretation of the mediation analyses assumes no unmeasured confounding with respect to (i) exposure–outcome, ii) mediator–outcome or (iii) exposure–mediator and (4) no mediator–outcome confounder affected by the exposure. The assumption of rare outcome (childhood cancer) was met for the use of logistic regression models. To address assumption (2), we adjusted for the following potential mediator–outcome confounders: birth-year, country and maternal age, and performed sensitivity analyses where we included IVF (in vitro fertilization) and maternal smoking as confounders (did not change the results, see Supplementary Table S10, available as Supplementary data at *IJE* online). Since sex was the exposure of interest in these analyses (with birth defects as a mediator), both assumptions (i) and (iii) regarding unmeasured confounding are plausible. Assumption (iv) is likely also fulfilled based on current knowledge. Results shown only for the cancer types for which there was a sex effect, for full table see Supplementary Table S11 (available as Supplementary data at *IJE* online).

b The CDE is the effect of sex (with females as reference) not mediated through birth defects (estimated for no birth defect).

c The NIE captures the portion of the sex effect explained by birth defect mediation alone.

d The NDE compares cancer risk in males with that in females if birth defect status for males was set to what would have been observed had they been females.

e Percentage mediated not calculated when the NDE and NIE were in opposite directions or when the CI for NIE contained the null.

### Sensitivity analyses

When leaving out one country at a time, we observed small differences from the results displayed in Figures 1 and 2 and Tables 2–4. Leaving out Finland resulted in slightly lower ORs, as expected due to the younger population. Additional sensitivity analyses including only children born 1990 onwards yielded similar results, with slightly higher ORs due to the younger population (see Supplementary sensitivity analyses—Description of results, available as Supplementary data at *IJE* online).

## Discussion

This large Nordic population-based study showed an increased risk of cancer among children with birth defects, with a greater risk among children with chromosomal compared with non-chromosomal birth defects. Among children with non-chromosomal birth defects, the strongest association was observed between neural tube defects and intracranial and intraspinal germ cell tumours. For chromosomal birth defects, the strongest association was seen between Down syndrome and AML. The birth defect–cancer associations were generally stronger among females than males with sex–birth defect interactions for any birth defect and overall cancer, non-chromosomal birth defects and germ cell tumours, non-chromosomal birth defects and lymphomas, and chromosomal birth defects and leukaemia. Sex was not a strong risk factor for childhood cancer, and mediation analysis suggested that only a relatively small percentage of the overall association between sex and childhood cancer was mediated through birth defects, although larger among the youngest children.

The major strengths of this study are the large number of cancer cases, classified according to ICCC-3, from population-based national registries with accurate and nearly complete information on cancer cases.^14^ Also, due to the national identification numbers, all individuals in the Nordic countries can be followed from birth till death, and there is little emigration. Whereas a limitation of the study is the lack of information on other possible confounders (e.g. parental income and education), there are no established risk factors associated strongly enough with both birth defects and cancer to explain our results. For an unmeasured confounder to explain the observed OR of 1.9 for any non-chromosomal birth defect and childhood cancer association, conditioned on the measured covariates, it would have to be associated with a 3-fold increased risk of both birth defects and childhood cancer (E-value for estimate E = 3.2, and E = 3.0 for lower confidence limit). In addition, multiple sensitivity analyses yielded stable results, supporting the main conclusions of the paper. There was limited statistical precision for specific combinations of birth defects and cancers, especially for analyses stratified by sex, and spurious associations from multiple comparisons could have resulted. Birth defect ascertainment has changed over time and among countries,^3^ but this would likely be random regarding a subsequent cancer diagnosis. Also, survival from birth defects has improved over time, and it is possible that this has been differential by sex. However, sensitivity analyses including only children born from 1990 indicate that these trends did not affect the results significantly. For the mediation analyses, non-differential misclassification of the mediator (birth defect), if present, would lead to underestimation of the NIE and overestimation of the NDE; hence the proportion mediated would be underestimated.

Our findings are consistent with previous studies that were smaller or had less complete data, whereas we included all cancer cases in the Nordic countries.^4^^,^^5^ Further, many of the observed specific birth defect–cancer associations agree with previous results, such as the risk of AML among children with Down syndrome and the risk of CNS tumours among children with nervous system defects. Also, the increasing risk by the numbers of defects and by younger age agrees with the literature.^4^^,^^5^

The biology underlying the association between birth defects and the risk of cancer later in life is poorly understood, but both genetic and environmental (epigenetic) factors are thought to be involved. One notion is that genetic abnormalities impairing normal development may predispose to both birth defects and malignancy. Large genome-wide association studies have, for instance, identified common genetic risk loci for orofacial clefts and co-occurring cancers.^28^ How epigenetics (DNA methylation) is involved in the aetiology of birth defects has been shown in individuals with orofacial clefts, displaying epigenome-wide hypomethylation compared with controls.^29^ In gene set enrichment analysis of oral cleft-associated differentially methylated regions, there was an over-representation of genes involved in the development of the palate^29^ which also are involved in tumour development, thus underscoring the association between birth defects and risk of cancer. Although we did not observe an association between orofacial clefts and cancer in our study, this has been reported before.^3–5^

Few studies have examined sex-specific differences in the association between birth defects and childhood cancer. Instead, they adjusted for sex. Yang *et al*. (1995)^12^ reported a 3-fold increase in the risk of rhabdomyosarcoma for males with birth defects but no increased risk for females, in contrast to our findings based on a larger number of cases (males: OR = 1.9, 1.1–3.2; females: OR = 2.1, 1.1–4.2). Johnson *et al*. (2009)^11^ reported an association between birth defects (including minor birth defects) and germ cell tumours for males (OR = 2.5, 95% CI 1.4–4.9) but not for females (1.1, 0.7–1.8). Based on a larger number of cases, we observed a similar risk estimate for germ cell tumours among males (2.0, 1.4–2.7) but an even higher risk among females (4.8, 3.3–6.9).

Different mechanisms may explain the male excess in both birth defects and childhood cancer, including genetic/chromosomal, environmental/epigenetic, hormonal and other biological factors. Studies have suggested aetiological heterogeneity by sex for childhood cancers for gestational age, maternal education, race/ethnicity and paternal age.^30^ Furthermore, sex differences in the immune system, hormonal milieu and dosage of the X chromosome may also play a role.^30–34^ As for childhood cancer, several studies have shown a male excess in birth defects, both overall and for most isolated birth defects with exceptions such as isolated cleft palate, choanal atresia and most neural tube defects (NTDs).^8^^,^^35–38^ Although the evidence for explaining the male-to-female sex ratio is scarce, several factors have been proposed. Interaction with sex has, for instance, been reported for the association between growth restriction and NTD, paternal age and cleft lip with or without cleft palate, and multigravidity and postaxial polydactyly as well as spina bifida without hydrocephalus.^36^ A higher prenatal mortality in male fetuses with birth defects may also influence the observed sex ratio at birth.

In contrast to the male excess in both birth defects and childhood cancer in our study, the birth defect–cancer association was in general stronger in females. The reason for this is unclear but likely involves a multitude of interactions between sex-specific factors and gene networks both pre- and postnatally.^39^

Marcotte *et al*. (2020)^13^ recently proposed that birth defects are a strong mediator for the association between sex and childhood cancer and noted large variations in the proportion mediated across cancer types and age at diagnosis. On the contrary, our data suggest that the proportions mediated by birth defects are smaller. For instance, whereas they estimated that 38% of the risk of any childhood cancer (0–18 years) was mediated by birth defects, we estimated 5% (0–19 years). Among children below 1 year of age they estimated 85% and we estimated 28%. Like Marcotte *et al*.,^13^ we observed an NIE for extracranial germ cell tumours and an inverse association for the NDE, also for renal tumours and leukaemia among children diagnosed before the age of one, indicating that the observed sex effect would have been stronger in the absence of an effect of birth defects. The greater proportion of children with birth defects in the study of Marcotte *et al*.^13^ (14.1% among cancer cases and 5.3% among births without cancer) than in our study (5.1% among cancer cases and 2.2% among controls) may partly explain the different findings. Only 70% of their cancer cases were successfully linked to birth certificates and included in the study population, whereas 95% of the children with birth defects were included, which could have introduced selection bias. The availability of information on potential confounders varied between the studies, but this is unlikely to explain the differences in results.

## Conclusion

Overall, our study showed an increased cancer risk among individuals with birth defects, and sex differences for some birth defect–cancer associations, with stronger associations among females. Further, we found that only a small proportion of the association between sex and childhood cancer was explained by birth defects, although higher among the youngest, suggesting that most of the association between sex and childhood cancer risk operates through other pathways. Our findings contribute new knowledge about sex differences in the association between birth defects and childhood cancer and suggest further research into the underlying mechanisms.

## Ethics approval

The study was approved by ethics committees in Norway (2015/317/REK vest) and Stockholm, Sweden (2015/1642–31/2), and by the Data Protection Agency in Denmark (2015–57-0002). Permission to use health register data in Finland was granted by the Finnish Institute of Health and Welfare (THL/68/5.05/2014 and THL/909/5.05/2015) after consultation with the country's data protection authority.

## Supplementary Material

dyac192_Supplementary_Data

## Data Availability

The datasets analysed during the current study are not freely available due to national regulations, but similar data can be obtained from the register authorities.
