# Correction to: Sex differences in childhood cancer risk among children with major birth defects: a Nordic population-based nested case-control study

**DOI:** 10.1093/ije/dyad067

**Published:** 2023-05-15

This is a correction to: Dagrun Slettebø Daltveit, Kari Klungsøyr, Anders Engeland, Anders Ekbom, Mika Gissler, Ingrid Glimelius, Tom Grotmol, Laura Madanat-Harjuoja, Anne Gulbech Ording, Henrik Toft Sørensen, Rebecca Troisi, Tone Bjørge, Sex differences in childhood cancer risk among children with major birth defects: a Nordic population-based nested case-control study, *International Journal of Epidemiology*, dyac192, https://doi.org/10.1093/ije/dyac192

In Figure 2, which has three parts, the first part was missing and the third part was included twice.

The paper has been corrected.

