# Supplementary material for: Sex differences in childhood cancer risk among children with major birth defects: a Nordic population-based nested case-control study
Source: Int J Epidemiol. 2022 Sep 30;52(2):450–65. doi: 10.1093/ije/dyac192 (PMC10114053; doi:10.1093/ije/dyac192)
Supplement: dyac192_Supplementary_Data [file dyac192_supplementary_data.pdf]

Supplemental content

Supplementary Table S1. Description of the registries. 2

Supplementary Table S2. Male-to-female sex ratios for the main cancer groups in the total study population, and associations between male sex and any or specific cancers. 3

Supplementary Table S3. Sex distribution [N (per 10 000) for males and females, and M:F sex ratios] for the major anomaly groups in the total study population. 6

Supplementary Table S4. Sensitivity analyses: adjusting for maternal age. 7

Supplementary Table S5. Sensitivity analyses: adjusting for maternal age and smoking. 8

Supplementary Table S6. Sensitivity analyses: adjusting for maternal age, smoking and IVF. 8

Supplementary Table S7. Association between Down syndrome and leukaemia by age at diagnosis. .. 9

Supplementary Table S8. Risk of any cancer in children with major birth defects stratified by sex and age at diagnosis. 9

Supplementary Table S9. Mediation analyses of the effect of non–chromosomal birth defects on the association between sex (males versus females) and childhood cancer, overall and by cancer site. 10

Supplementary Table S10. Sensitivity analyses for the mediation analyses. 10

Supplementary Table S11. Mediation analyses of the effect of birth defects on the association between sex (males versus females) and childhood cancer, overall and by cancer site, complete table. 11

Supplementary Table S12. Risk of specific cancers in individuals with selected specific birth defects. 13

Supplementary Figure S1. A simplified illustration of the assumed causal relationship between sex, birth defects and childhood cancer 19

Supplementary Figure S2. Risk of cancer according to number of major birth defects (1, 2 or more) in different anatomical subgroups. 20

Supplementary Figure S3. Risk of cancer in children with major birth defects, stratified by age at cancer diagnosis. 21

Supplementary sensitivity analyses – Description of results 22

Supplementary Table S1. Description of the registries.

Registry	Country	Description	Coding	Supporting references
The Medical Birth Registries	All countries	Contains information on all births in Denmark, Finland, Norway, and Sweden since 1973, 1987, 1967, and 1973, respectively.	Denmark: 1973-1993: ICD-8, 1994 onwards: ICD-10.<br>Finland:1987-1995: ICD-9, 1996 onwards: ICD-10.<br>Norway: 1967-1998: ICD-8 (with some internally generated codes), 1999 onwards: ICD-10.<br>Sweden: 1973-1986: the Swedish versions of ICD-8, 1987-1996: ICD-9, 1997 onwards: ICD-10.	Langhoff-Roos J, Krebs L, Klungsoyr K, *et al*. The Nordic medical birth registers--a potential goldmine for clinical research. Acta obstetricia et gynecologica Scandinavica. 2014;93(2):132-7.
The National Patient Registries	Denmark and Sweden	Administrative nationwide registries on inpatient care since 1978 in Denmark and 1987 in Sweden.	Same as in the Birth Registries.	Schmidt M, Schmidt SA, Sandegaard JL, *et al*. The Danish National Patient Registry: a review of content, data quality, and research potential. Clin Epidemiol 2015;7:449-90.<br><br>Ludvigsson JF, Andersson E, Ekbom A, *et al*. External review and validation of the Swedish national inpatient register. BMC Public Health 2011;11:450.
The Register of Congenital Malformations	Finland	Information on birthdefects on live and stillborn. since 1963.	1986 onwards: ICD-9 Atlanta modification, retrospective inclusion of ICD-10 codes from 1996.	Ritvanen A. Epämuodostumat 1993-2011 – Congenital anomalies 1993-2011. National Institute for Health and Welfare in Finland, 2014.
The Cancer Registries	All countries	Covers the entire populations in Denmark, Finland, Norway, and Sweden since 1943, 1953, 1953, and 1958, respectively.	All countries currently provides ICD-O-3 codes. Older cancer cases coded with older ICD versions, see Pukkala *et al*. (2018).	Pukkala E, Engholm G, Hojsgaard Schmidt LK *et al*. Nordic Cancer Registries – an overview of their procedures and data comparability. Acta oncologica (Stockholm, Sweden). 2018;57(4):440-55.
The National Population Registries.	All countries	Administrative registry of the whole population in Denmark, Finland, Norway, and Sweden since 1968, 1971, 1964, and 1968, respectively. Contains information on deaths and emigration.	NA	Laugesen K, Ludvigsson JF, Schmidt M, *et al*. Nordic Health Registry-Based Research: A Review of Health Care Systems and Key Registries. Clinical epidemiology 2021; 13: 533-54.

Supplementary Table S2. Male-to-female sex ratios for the main cancer groups in the total study population, and associations between male sex and any or specific cancers.

Cancer site[a]	N cases	M:F sex ratio	OR	95% CI
Any cancer	21,898	1.14	1.16	(1.13–1.19)
I Leukaemia, myeloproliferative and myelodysplastic diseases	5552	1.17	1.16	(1.10–1.23)
(a) Lymphoid leukaemia	4156	1.18	1.18	(1.11–1.25)
(a.1) Precursor cell leukaemia	4089	1.17	1.17	(1.10–1.24)
(a.2) Mature B-cell leukaemia	45	2.14	2.13	(1.13–4.01)
(a.3) Mature T-cell and NK cell leukaemia	8	6.78	6.57	(0.81–53.4)
(a.4) Lymphoid leukaemia, NOS	14	1.29	1.30	(0.45–3.74)
(b) Acute myeloid leukaemia	885	1.03	1.03	(0.90–1.17)
(c) Chronic myeloproliferative diseases	173	1.18	1.19	(0.88–1.60)
(d) Myelodysplastic syndrome and other myeloproliferative diseases	103	1.30	1.26	(0.85–1.87)
(e) Unspecified and other specified leukaemia	235	1.45	1.49	(1.15–1.93)
II Lymphomas and reticuloendothelial neoplasms	2907	1.49	1.51	(1.41–1.63)
(a) Hodgkin lymphomas	1438	1.06	1.08	(0.97–1.20)
(b) Non-Hodgkin lymphomas (except Burkitt lymphoma)	987	1.89	1.92	(1.68–2.19)
(b.1) Precursor cell lymphomas	186	2.43	2.39	(1.74–3.29)
(b.2) Mature B-cell lymphomas (except Burkitt lymphoma)	280	1.88	1.92	(1.50–2.45)
(b.3) Mature T-cell and NK-cell lymphomas	177	1.59	1.57	(1.16–2.13)
(b.4) Non-Hodgkin lymphomas, NOS	344	1.83	1.93	(1.54–2.41)
(c) Burkitt lymphoma	292	4.36	4.32	(3.21–5.82)
(d) Miscellaneous lymphoreticular neoplasms	131	1.79	1.72	(1.20–2.46)
(e) Unspecified lymphomas	59	1.75	1.73	(1.01–2.95)
III CNS and miscellaneous intracranial and intraspinal neoplasms	5177	1.14	1.14	(1.08–1.20)
(a) Ependymomas and choroid plexus tumour	514	1.36	1.35	(1.13–1.61)
(a.1) Ependymomas	397	1.29	1.29	(1.06–1.58)
(a.2) Choroid plexus tumour	117	1.61	1.58	(1.08–2.29)
(b) Astrocytoma	1967	1.04	1.04	(0.95–1.14)
(c) Intracranial and intraspinal embryonal tumours	958	1.56	1.55	(1.36–1.76)
(c.1) Medulloblastomas	637	1.64	1.64	(1.40–1.93)
(c.2) Primitive neuroectodermal tumour (PNET)	266	1.33	1.31	(1.03–1.68)
(c.3) Medulloepithelioma	12	0.97	0.94	(0.30–2.92)
(c.4) Atypical teratoid/rhabdoid tumour	43	2.23	2.14	(1.12–4.10)
(d) Other gliomas	469	0.93	0.95	(0.79–1.14)
(d.1) Oligodendrogliomas	125	1.08	1.08	(0.76–1.53)
(d.2) Mixed and unspecified gliomas	326	0.84	0.86	(0.69–1.06)
(d.3) Neuroepithelial glial tumours of uncertain origin	18	2.52	2.63	(0.94–7.38)
(e) Other specified intracranial and intraspinal neoplasms	789	1.07	1.07	(0.93–1.23)
(e.1) Pituitary adenomas and carcinomas	51	0.37	0.36	(0.19–0.66)
(e.2) Tumours of the sellar region (craniopharyngiomas)	232	0.97	0.96	(0.74–1.25)
(e.3) Pineal parenchymal tumours	57	0.76	0.76	(0.45–1.28)
(e.4) Neuronal and mixed neuronal-glial tumours	334	1.43	1.44	(1.15–1.79)
(e.5) Meningiomas	115	1.06	1.07	(0.74–1.54)
(f) Unspecified intracranial and intraspinal neoplasms	480	1.01	1.00	(0.83–1.19)
IV Neuroblastoma and other peripheral nervous cell tumours	1143	1.16	1.15	(1.03–1.30)
(a) Neuroblastoma and ganglioneuroblastoma	1118	1.15	1.14	(1.01–1.28)
(b) Other peripheral nervous cell tumours	25	2.06	2.07	(0.89–4.80)

Cancer site[a]	N cases	M:F sex ratio	OR	95% CI
V Retinoblastoma	435	1.13	1.12	(0.93–1.35)
VI Renal tumours	1012	0.89	0.88	(0.78–1.00)
(a) Nephroblastoma and other non-epithelial renal tumours	965	0.88	0.87	(0.77–0.99)
(a.1) Nephroblastoma	947	0.89	0.88	(0.78–1.00)
(a.2) Rhabdoid renal tumour	7	0.39	0.37	(0.07–1.91)
(a.3) Kidney sarcomas	11	0.81	0.79	(0.24–2.58)
(b) Renal carcinomas	38	1.48	1.51	(0.79–2.89)
(c) Unspecified malignant renal tumours	9	0.28	0.28	(0.06–1.33)
VII Hepatic tumours	291	1.42	1.40	(1.11–1.77)
(a) Hepatoblastoma and mesenchymal tumours of liver	199	1.53	1.50	(1.13–2.00)
(a.1) Hepatoblastoma	193	1.63	1.59	(1.19–2.13)
(a.3) Embryonal sarcoma of liver	5	0.24	0.23	(0.03–2.04)
(b) Hepatic carcinomas	90	1.16	1.17	(0.77–1.78)
VIII Malignant bone tumours	948	1.17	1.16	(1.02–1.32)
(a) Osteosarcomas	524	1.24	1.24	(1.04–1.47)
(b) Chondrosarcomas	48	0.97	0.97	(0.55–1.71)
(c) Ewing tumour and related sarcomas of bone	314	1.16	1.14	(0.92–1.43)
(c.1) Ewing tumour and Askin tumour of bone	310	1.16	1.15	(0.92–1.43)
(d) Other specified malignant bone tumours	40	0.79	0.79	(0.42–1.47)
(d.2) Malignant chordomas	25	0.89	0.88	(0.40–1.94)
(d.4) Miscellaneous malignant bone tumours	9	0.77	0.79	(0.21–2.94)
(e) Unspecified malignant bone tumours	22	1.16	1.16	(0.50–2.68)
IX Soft tissue and other extraosseous sarcomas	1335	1.23	1.23	(1.11–1.38)
(a) Rhabdomyosarcomas	571	1.32	1.32	(1.11–1.56)
(b) Fibrosarcoma, peripheral nerve sheath tumours, and other fibrous neoplasms	199	1.10	1.11	(0.84–1.46)
(b.1) Fibroblastic and myofibroblastic tumours	111	1.32	1.32	(0.91–1.93)
(b.2) Nerve sheath tumours	86	0.88	0.89	(0.58–1.35)
(d) Other specified soft tissue sarcomas	444	1.19	1.20	(1.00–1.45)
(d.1) Ewing tumour and Askin tumour of soft tissue	69	1.26	1.24	(0.77–2.00)
(d.2) Peripheral neuroectodermal tumour (pPNET) of soft tissue	34	1.38	1.45	(0.73–2.88)
(d.3) Extrarenal extrahepatic rhabdoid tumour	7	0.73	0.69	(0.15–3.08)
(d.4) Liposarcomas	21	0.39	0.38	(0.15–0.99)
(d.5) Fibrohistiocytic tumours	101	1.20	1.21	(0.82–1.79)
(d.6) Leiomyosarcomas	20	1.80	1.77	(0.71–4.43)
(d.7) Synovial sarcomas	124	1.30	1.30	(0.91–1.85)
(d.8) Blood vessel tumours	14	0.39	0.39	(0.12–1.24)
(d.9) Osseous and chondromatous neoplasms of soft tissue	13	1.13	1.14	(0.38–3.39)
(d.10) Alveolar soft parts sarcoma	7	0.16	0.16	(0.02–1.35)
(d.11) Miscellaneous soft tissue sarcomas	34	2.69	3.07	(1.43–6.59)
(e) Unspecified soft tissue sarcomas	119	1.21	1.20	(0.83–1.72)
X Germ cell tumours. trophoblastic tumours and neoplasms of gonads	1313	2.21	2.22	(1.98–2.50)
(a) Intracranial and intraspinal germ cell tumours	204	1.98	1.98	(1.48–2.65)
(a.1) Intracranial and intraspinal germinomas	116	2.33	2.35	(1.57–3.50)
(a.2) Intracranial and intraspinal teratomas	64	1.33	1.31	(0.80–2.16)
(a.3) Intracranial and intraspinal embryonal carcinomas	5	3.87	3.84	(0.43–34.3)
(a.4) Intracranial and intraspinal yolk sac tumour	6	0.97	0.95	(0.19–4.70)

Cancer site[a]	N cases	M:F sex ratio	OR	95% CI
(a.6) Intracranial and intraspinal tumours of mixed forms	12	4.84	4.87	(1.07–22.0)
(b) Malignant extracranial and extragonadal germ cell tumours	146	0.66	0.65	(0.47–0.91)
(b.2) Malignant teratomas of extracranial and extragonadal sites	95	0.49	0.48	(0.32–0.74)
(b.3) Embryonal carcinomas of extracranial and extragonadal sites	9	1.21	1.23	(0.33–4.58)
(b.4) Yolk sac tumour of extracranial and extragonadal sites	27	0.67	0.67	(0.31–1.44)
(c) Malignant gonadal germ cell tumours	854	3.79	3.82	(3.23–4.52)
(c.1) Malignant gonadal germinomas	117	1.21	1.20	(0.84–1.73)
(c.2) Malignant gonadal teratomas	254	2.44	2.44	(1.86–3.21)
(c.3) Gonadal embryonal carcinomas	165	52.21	51.90	(16.6–163)
(c.4) Gonadal yolk sac tumour	123	2.34	2.35	(1.59–3.47)
(c.5) Gonadal choriocarcinoma	30	8.71	8.64	(2.62–28.5)
(c.6) Malignant gonadal tumours of mixed forms	165	21.82	22.00	(10.3–46.9)
(d) Gonadal carcinomas	49	0.04	0.04	(0.01–0.17)
(e) Other and unspecified malignant gonadal tumours	60	1.36	1.34	(0.80–2.24)
XI Other malignant epithelial neoplasms and malignant melanomas	1652	0.53	0.53	(0.48–0.59)
(a) Adrenocortical carcinomas	29	0.68	0.68	(0.32–1.42)
(b) Thyroid carcinomas	420	0.30	0.30	(0.24–0.38)
(c) Nasopharyngeal carcinomas	34	2.02	1.99	(0.97–4.08)
(d) Malignant melanomas	605	0.53	0.53	(0.45–0.63)
(e) Skin carcinomas	28	0.97	0.97	(0.46–2.04)
(f) Other and unspecified carcinomas	536	0.67	0.69	(0.58–0.82)
(f.1) Carcinomas of salivary glands	106	0.35	0.35	(0.23–0.54)
(f.2) Carcinomas of colon and rectum	74	0.66	0.75	(0.47–1.19)
(f.3) Carcinomas of appendix	161	0.61	0.63	(0.46–0.86)
(f.4) Carcinomas of lung	48	1.61	1.62	(0.90–2.9)
(f.6) Carcinomas of breast	10	0.11	0.11	(0.01–0.85)
(f.8) Carcinomas of bladder	7	2.42	3.06	(0.59–15.9)
(f.10) Carcinomas of other specified sites	100	1.14	1.15	(0.77–1.70)
(f.11) Carcinomas of unspecified site	19	0.56	0.56	(0.22–1.43)
XII Other and unspecified malignant neoplasms	133	0.68	0.68	(0.48–0.96)
(a) Other specified malignant tumours	26	0.43	0.43	(0.19–0.99)
(a.3) Pulmonary blastoma and pleuropulmonary blastoma	9	0.77	0.74	(0.20–2.75)
(a.4) Other complex mixed and stromal neoplasms	7	0.39	0.41	(0.08–2.11)
(a.5) Mesothelioma	5	0.24	0.25	(0.03–2.23)
(b) Other unspecified malignant tumours	107	0.76	0.76	(0.52–1.11)

[a]Sites with < 5 cases or with no female cases are not included. ORs adjusted for matching variables (birth year and country). Cancers classified in ICCC-3 groups.
Abbreviations: OR, odds ratio; CI, confidence interval; NOS, not otherwise specified; CNS, central nervous system.

Supplementary Table S3. Sex distribution [N (per 10 000) for males and females, and M:F sex ratios] for the major anomaly groups in the total study population.

Major birth defects	N (per 10 000) boys	N (per 10 000) girls	M:F Sex ratio
All anomalies	281	215	1.30
All anomalies excluding chromosomal anomalies	260	193	1.35
Nervous system	14	14	1.00
Neural tube defects	4	5	0.81
Eye	8	7	1.05
Ear, face, and neck	2	2	1.14
Congenital heart defects	70	76	0.91
Respiratory system	3	5	0.70
Oro-facial clefts	21	16	1.35
Cleft palate only	6	9	0.67
Cleft lip with/without cleft palate	16	7	2.25
Digestive system	15	16	0.97
Abdominal wall defects	3	2	1.14
Urinary system	22	12	1.82
Genital organs	44	5	8.93
Limb	50	34	1.49
Skeletal dysplasia	3	3	1.22
Genetic syndromes and microdeletions	6	6	1.03
Chromosomal	21	23	0.90
Down syndrome	17	19	0.88
Other anomalies/syndromes	41	32	1.29

Supplementary Table S4. Sensitivity analyses: adjusting for maternal age.

Cancer site	OR	95% CI
Non-chromosomal birth defects		
Any cancer	1.9	(1.8-2.0)
I Leukaemia, myeloproliferative and myelodysplastic diseases	1.2	(1.0-1.4)
II Lymphomas and reticuloendothelial neoplasms	1.5	(1.2-1.8)
III CNS and miscellaneous intracranial and intraspinal neoplasms	2.3	(2.0-2.6)
IV Neuroblastoma and other peripheral nervous cell tumours	2.7	(2.1-3.5)
V Retinoblastoma	1.1	(0.6-2.1)
VI Renal tumours	3.6	(2.8-4.6)
VII Hepatic tumours	3.0	(1.8-4.8)
VIII Malignant bone tumours	1.3	(0.9-2.0)
IX Soft tissue and other extraosseous sarcomas	2.2	(1.7-2.8)
X Germ cell tumours, trophoblastic tumours, and neoplasms of gonads	2.6	(2.1-3.4)
XI Other malignant epithelial neoplasms and malignant melanomas	1.7	(1.3-2.2)
XII Other and unspecified malignant neoplasms	3.2	(1.6-6.3)
Chromosomal birth defects		
Any cancer	9.6	(8.1-11)
I Leukaemia, myeloproliferative and myelodysplastic diseases	29.3	(24-35)
II Lymphomas and reticuloendothelial neoplasms	1.4	(0.6-3.5)
III CNS and miscellaneous intracranial and intraspinal neoplasms	1.9	(1.1-3.4)
IV Neuroblastoma and other peripheral nervous cell tumours	1.4	(0.3-5.6)
V Retinoblastoma	7.1	(2.6-19)
VI Renal tumours	5.0	(2.2-11)
VII Hepatic tumours	2.6	(0.4-19)
VIII Malignant bone tumours	1.0	(0.1-7.1)
IX Soft tissue and other extraosseous sarcomas	1.9	(0.6-6.1)
X Germ cell tumours, trophoblastic tumours, and neoplasms of gonads	4.2	(1.9-9.5)
XI Other malignant epithelial neoplasms and malignant melanomas	0.5	(0.1-3.7)
XII Other and unspecified malignant neoplasms	11.0	(2.7-45)

Abbreviations: OR, odds ratio; CI, confidence interval.

Supplementary Table S5. Sensitivity analyses: adjusting for maternal age and smoking.

	Adjusted* for maternal age		Adjusted* for maternal age and smoking	
Cancer site	OR	95% CI	OR	95% CI
Non-chromosomal birth defects				
Any cancer	2.1	(1.9-2.2)	2.1	(1.9-2.3)
I Leukaemia, myeloproliferative and myelodysplastic diseases	1.2	(1.0-1.5)	1.3	(1.1-1.6)
II Lymphomas and reticuloendothelial neoplasms	1.8	(1.4-2.2)	1.8	(1.4-2.3)
III CNS and miscellaneous intracranial and intraspinal neoplasms	2.5	(2.2-2.9)	2.5	(2.2-2.9)
IV Neuroblastoma and other peripheral nervous cell tumours	2.9	(2.2-3.8)	2.9	(2.2-3.9)
V Retinoblastoma	1.0	(0.5-2.2)	1.1	(0.5-2.4)
VI Renal tumours	4.1	(3.2-5.3)	4.2	(3.2-5.5)
VII Hepatic tumours	3.7	(2.2-6.1)	3.7	(2.2-6.2)
VIII Malignant bone tumours	1.3	(0.8-2.2)	1.4	(0.9-2.4)
IX Soft tissue and other extraosseous sarcomas	2.6	(2.0-3.5)	2.6	(2.0-3.5)
X Germ cell tumours, trophoblastic tumours, and neoplasms of gonads	3.2	(2.4-4.2)	3.1	(2.3-4.1)
XI Other malignant epithelial neoplasms and malignant melanomas	1.4	(1.0-2.0)	1.4	(1.0-2.0)
XII Other and unspecified malignant neoplasms	3.2	(1.5-6.9)	3.5	(1.6-7.5)
Chromosomal birth defects.				
Any cancer	10.5	(8.7-13)	10.7	(8.7-13)
I Leukaemia, myeloproliferative and myelodysplastic diseases	29.7	(24.1-37)	29.6	(24-37)
II Lymphomas and reticuloendothelial neoplasms	1.9	(0.8-4.7)	2.1	(0.9-5.1)
III CNS and miscellaneous intracranial and intraspinal neoplasms	2.5	(1.4-4.4)	2.7	(1.5-4.8)
IV Neuroblastoma and other peripheral nervous cell tumours	1.8	(0.5-7.4)	1.9	(0.5-7.7)
V Retinoblastoma	7.2	(2.3-23)	7.7	(2.4-24)
VI Renal tumours	5.2	(2.1-13)	5.5	(2.2-13)
VII Hepatic tumours	3.5	(0.5-25)	3.9	(0.5-28)
VIII Malignant bone tumours	1.5	(0.2-11)	1.6	(0.2-11)
IX Soft tissue and other extraosseous sarcomas	2.6	(0.8-8.2)	2.8	(0.9-8.8)
X Germ cell tumours, trophoblastic tumours, and neoplasms of gonads	4.2	(1.6-11)	4.6	(1.7-12)
XI Other malignant epithelial neoplasms and malignant melanomas	0.7	(0.1-5.1)	0.8	(0.1-5.5)
XII Other and unspecified malignant neoplasms	14.9	(3.6-62)	16.3	(3.9-68)

*Also adjusted for matching variables (country and birth year) and sex. Abbreviations: OR, odds ratio; CI, confidence interval, CNS, central nervous system.

Supplementary Table S6. Sensitivity analyses: adjusting for maternal age, smoking and IVF.

	OR (95%CI) Adjusted for country, birth year, and sex	OR (95%CI) Adjusted for country, birth year, sex, and IVF	OR (95%CI) Adjusted for country, birth year, sex, IVF, and maternal age
Non-chromosomal birth defects	2.1 (1.9-2.4)	2.1 (1.9-2.3)	2.1 (1.9-2.3)
Chromosomal birth defects	11.8 (9.5-14.7)	11.8 (9.5-14.7)	10.7 (8.5-13.3)

Abbreviations: OR, odds ratio; CI, confidence interval; IVF, *in vitro* fertilization.

Supplementary Table S7. Association between Down syndrome and leukaemia by age at diagnosis.

Birth defect	*Cancer*	*Age at diagnosis*	*OR (95% CI)*	*n cases*	*n (%) cases with BD*
Down Syndrome	ALL	<2 year	12 (5.4-28)	455	6 (1.3)
Down Syndrome	ALL	2-4 years	22 (15-31)	1 714	38 (2.2)
Down Syndrome	ALL	5+ years	27 (20-37)	1871	46 (2.5)
Down Syndrome	AML	<1 year	253 (155-413)	110	24 (22)
Down Syndrome	AML	1 year	451 (305-667)	143	45 (31)
Down Syndrome	AML	2-4 years	256 (170-387)	163	33 (20)
Down Syndrome	AML	5+ years	7.7 (2.4-24)	450	3 (0.7)

Abbreviations: OR, odds ratio; CI, confidence interval; BD, birth defect; ALL, acute lymphoid leukaemia; AML, acute myeloid leukaemia.

Supplementary Table S8. Risk [OR (95% CI)] of any cancer in children with major birth defects stratified by sex and age at diagnosis.

	0-4 years		5-9 years		10-14 years		15-19 years	
	Males	Females	Males	Females	Males	Females	Males	Females
All anomalies	2.7 (2.4-3.1)	4.1 (3.6-4.6)	1.7 (1.4-2.1)	2.2 (1.7-2.8)	1.8 (1.5-2.3)	2.0 (1.5-2.5)	1.4 (1.2-1.7)	1.8 (1.4-2.3)
All anomalies excl. chromosomal	2.1 (1.9-2.4)	2.8 (2.4-3.3)	1.4 (1.1-1.8)	1.9 (1.5-2.5)	1.6 (1.3-2.0)	1.8 (1.3-2.4)	1.4 (1.1-1.7)	1.7 (1.4-2.2)

Abbreviations: OR, odds ratio; CI, confidence interval.

Supplementary Table S9. Mediation analyses of the effect of non–chromosomal birth defects on the association between sex (males versus females) and childhood cancer, overall and by cancer site.

	Controlled direct effect[a] OR (95% CI)	Natural indirect effect OR (95% CI)	Natural direct effect OR (95% CI)	Marginal total effect OR (95% CI)	Percentage (%) mediated[b]
**Total study population 0–19 years**					
Any cancer	1.17 (1.14–1.20)	1.005 (1.003–1.006)	1.16 (1.13–1.19)	1.17 (1.13–1.20)	3.29
I Leukaemia, myeloproliferative and myelodysplastic diseases	1.19 (1.12–1.25)	1.001 (0.999–1.003)	1.19 (1.12–1.25)	1.19 (1.12–1.25)	..
II Lymphomas and reticuloendothelial neoplasms	1.53 (1.42–1.65)	1.002 (0.999–1.004)	1.52 (1.41–1.63)	1.52 (1.41–1.64)	..
III CNS and miscellaneous intracranial and intraspinal neoplasms	1.14 (1.08–1.21)	1.007 (1.004–1.010)	1.13 (1.07–1.19)	1.14 (1.08–1.20)	5.70
IV Neuroblastoma and other peripheral nervous cell tumours	1.16 (1.03–1.31)	1.009 (1.004–1.015)	1.15 (1.02–1.29)	1.16 (1.03–1.30)	6.80
V Retinoblastoma	1.12 (0.93–1.36)	0.999 (0.994–1.005)	1.11 (0.92–1.35)	1.11 (0.92–1.34)	..
VI Renal tumours	0.90 (0.79–1.02)	1.014 (1.007–1.021)	0.88 (0.78–1.00)	0.89 (0.79–1.01)	..
VII Hepatic tumours	1.44 (1.13–1.84)	1.010 (0.999–1.020)	1.41 (1.11–1.78)	1.42 (1.12–1.80)	..
VIII Malignant bone tumours	1.16 (1.02–1.32)	1.002 (0.999–1.006)	1.16 (1.02–1.31)	1.16 (1.02–1.32)	..
IX Soft tissue and other extraosseous sarcomas	1.22 (1.09–1.36)	1.008 (1.003–1.013)	1.23 (1.10–1.37)	1.24 (1.11–1.38)	4.33
X Germ cell tumours, trophoblastic tumours, and neoplasms of gonads	2.30 (2.04–2.60)	1.006 (1.002–1.010)	2.19 (1.95–2.47)	2.21 (1.96–2.48)	1.13
XI Other malignant epithelial neoplasms and malignant melanomas	0.53 (0.48–0.59)	1.004 (1.000–1.009)	0.53 (0.48–0.59)	0.53 (0.48–0.59)	..
XII Other and unspecified malignant neoplasms	0.68 (0.47–0.97)	1.016 (0.996–1.036)	0.67 (0.48–0.95)	0.68 (0.48–0.97)	..
**Children younger than 5 years at time of diagnosis**					
Any cancer	1.13 (1.08–1.18)	1.007 (1.005–1.010)	1.11 (1.07–1.17)	1.12 (1.07–1.17)	6.71
**Children younger than 1 year at time of diagnosis**					
Any cancer	1.10 (0.99–1.21)	1.021 (1.015–1.029)	1.07 (0.97–1.18)	1.09 (0.99–1.20)	24.82

[a]Estimated for no birth defect. [b]Proportion mediated not calculated when the NDE and NIE were in opposite directions or when the CI for NIE contained the null effect. Abbreviations: OR, odds ratio; CI, confidence interval.

Supplementary Table S10. Sensitivity analyses for the mediation analyses.

	Controlled direct effect[a] OR (95% CI)	Natural indirect effect OR (95% CI)	Natural direct effect OR (95% CI)	Marginal total effect OR (95% CI)	Percentage (%) mediated
Restricted to countries and time period when IVF was reported[b].	1.11 (1.07-1.16)	1.009 (1.007-1.011)	1.11 (1.07-1.16)	1.12 (1.08-1.17)	8.27
Including IVF as a confounder.	1.11 (1.07-1.16)	1.009 (1.007-1.011)	1.11 (1.07-1.16)	1.12 (1.08-1.17)	8.27
Restricted to time period when maternal smoking was reported[c].	1.14 (1.10-1.18)	1.011 (1.009-1.013)	1.14 (1.10-1.18)	1.15 (1.11-1.20)	7.93
Including maternal smoking as a confounder.	1.14 (1.10-1.18)	1.011 (1.009-1.013)	1.14 (1.10-1.18)	1.15 (1.11-1.20)	7.93

[a]Estimated for no birth defect. [b]Median age at diagnosis in this subpopulation was 5 years (compared to 8 years in the total study population). [c]Median age at diagnosis in this sub population was 6 years. Abbreviations: OR, odds ratio; CI, confidence interval; IVF, *in vitro* fertilization.

Supplementary Table S11. Mediation analyses of the effect of birth defects on the association between sex (males versus females) and childhood cancer, overall and by cancer site, complete table.

Cancer site	Controlled direct effect[a] OR (95% CI)	Natural indirect effect OR (95% CI)	Natural direct effect OR (95% CI)	Marginal total effect OR (95% CI)	Percentage (%) mediated[b]
**Total study population 0–19 years**					
Any cancer	1.17 (1.14–1.20)	1.007 (1.005–1.008)	1.16 (1.12–1.19)	1.16 (1.13–1.20)	4.80
I Leukaemia, myeloproliferative, and myelodysplastic diseases	1.19 (1.12–1.25)	1.009 (1.006–1.012)	1.16 (1.10–1.22)	1.17 (1.11–1.24)	5.95
(a) Lymphoid leukaemia	1.19 (1.12–1.27)	1.005 (1.003–1.008)	1.18 (1.11–1.25)	1.18 (1.11–1.26)	3.42
(b) Acute myeloid leukaemia	1.06 (0.92–1.23)	1.028 (1.018–1.038)	1.00 (0.88–1.15)	1.03 (0.90–1.18)	85.44
Other leukaemia	1.33 (1.11–1.60)	1.008 (1.001–1.015)	1.32 (1.11–1.57)	1.33 (1.11–1.58)	3.16
II Lymphomas and reticuloendothelial neoplasms	1.53 (1.42–1.66)	1.002 (1.000–1.004)	1.52 (1.41–1.63)	1.52 (1.41–1.64)	..
(a) Hodgkin lymphomas	1.09 (0.98–1.22)	0.998 (0.996–1.001)	1.08 (0.97–1.20)	1.08 (0.97–1.20)	..
(b) Non–Hodgkin lymphomas	1.95 (1.71–2.24)	1.004 (1.000–1.009)	1.92 (1.68–2.19)	1.93 (1.69–2.20)	0.92
Other lymphomas	2.92 (2.37–3.61)	1.003 (0.998–1.009)	2.88 (2.34–3.54)	2.89 (2.35–3.55)	..
III CNS and miscellaneous intracranial and intraspinal neoplasms	1.14 (1.08–1.21)	1.007 (1.004–1.010)	1.13 (1.08–1.21)	1.14 (1.08–1.20)	5.69
(a.1) Ependymomas	1.30 (1.06–1.59)	1.000 (0.995–1.006)	1.29 (1.06–1.58)	1.29 (1.06–1.58)	..
(b) Astrocytoma	1.05 (0.96–1.15)	1.005 (1.001–1.008)	1.04 (0.95–1.13)	1.04 (0.95–1.14)	12.27
(c.1) Medulloblastomas	1.70 (1.44–2.00)	1.000 (0.996–1.004)	1.64 (1.40–1.93)	1.64 (1.40–1.93)	..
(c.2) Primitive neuroectodermal tumour	1.36 (1.06–1.74)	1.003 (0.995–1.011)	1.31 (1.03–1.68)	1.32 (1.03–1.68)	..
Other CNS	1.03 (0.94–1.14)	1.014 (1.009–1.019)	1.04 (0.95–1.14)	1.06 (0.97–1.16)	24.79
IV Neuroblastoma and other peripheral nervous cell tumours	1.16 (1.03–1.31)	1.009 (1.004–1.014)	1.15 (1.02–1.29)	1.16 (1.03–1.30)	6.52
(a) Neuroblastoma	1.14 (1.01–1.29)	1.009 (1.004–1.014)	1.13 (1.01–1.27)	1.14 (1.01–1.29)	7.08
(b) Other peripheral nervous cell tumours	2.25 (0.93–5.47)	1.010 (0.978–1.042)	2.05 (0.88–4.77)	2.07 (0.89–4.81)	..
V Retinoblastoma	1.12 (0.93–1.36)	1.002 (0.996–1.009)	1.12 (0.93–1.35)	1.12 (0.93–1.35)	..
VI Renal tumours	0.90 (0.79–1.02)	1.013 (1.006–1.020)	0.88 (0.77–0.99)	0.89 (0.78–1.00)	..
(a.1) Nephroblastoma	0.90 (0.78–1.02)	1.013 (1.006–1.021)	0.87 (0.77–0.99)	0.88 (0.78–1.00)	..
Other renal	0.93 (0.57–1.51)	1.003 (0.990–1.016)	0.93 (0.57–1.51)	0.93 (0.57–1.51)	..
VII Hepatic tumours	1.44 (1.13–1.84)	1.009 (0.999–1.018)	1.40 (1.11–1.77)	1.41 (1.12–1.78)	2.94
(a.1) Hepatoblastoma	1.69 (1.25–2.29)	1.006 (0.996–1.017)	1.59 (1.19–2.13)	1.60 (1.19–2.14)	..
Other hepatic	1.09 (0.73–1.65)	1.014 (0.994–1.035)	1.11 (0.73–1.65)	1.13 (0.76–1.68)	..
VIII Malignant bone tumours	1.16 (1.02–1.32)	1.002 (0.999–1.006)	1.16 (1.02–1.32)	1.17 (1.03–1.33)	..
(a) Osteosarcoma	1.25 (1.05–1.49)	1.002 (0.996–1.007)	1.23 (1.04–1.47)	1.24 (1.04–1.47)	..
(c.1) Ewing tumour and Askin tumour of bone	1.12 (0.90–1.41)	1.003 (0.996–1.011)	1.14 (0.91–1.43)	1.15 (0.92–1.44)	..
Other bone	0.93 (0.65–1.35)	1.003 (0.992–1.013)	0.93 (0.65–1.35)	0.93 (0.65–1.35)	..
IX Soft tissue and other extraosseous sarcomas	1.22 (1.09–1.36)	1.008 (1.003–1.013)	1.22 (1.10–1.37)	1.23 (1.11–1.38)	4.18
(a) Rhabdomyosarcoma	1.31 (1.11–1.56)	1.005 (0.999–1.011)	1.31 (1.11–1.55)	1.32 (1.12–1.56)	..
Other soft tissue	1.16 (1.00–1.34)	1.010 (1.003–1.017)	1.16 (1.01–1.34)	1.18 (1.02–1.36)	6.68
X Germ cell tumours, trophoblastic tumours and neoplasms of gonads	2.30 (2.04–2.60)	1.007 (1.003–1.012)	2.21 (1.96–2.48)	2.22 (1.97–2.50)	1.33
(a) Intracranial germ cell tumours	2.09 (1.53–2.84)	1.015 (1.002–1.029)	1.95 (1.45–2.61)	1.98 (1.48–2.65)	3.08
(b) Extracranial germ cell tumours	0.68 (0.48–0.97)	1.025 (1.002–1.048)	0.64 (0.46–0.90)	0.66 (0.47–0.92)	..
(c), (d), and (e) Gonadal germ cell tumours	2.86 (2.47–3.32)	1.004 (1.000–1.009)	2.83 (2.45–3.28)	2.85 (2.46–3.29)	0.67
XI Other malignant epithelial neoplasms and malignant melanomas	0.53 (0.48–0.59)	1.004 (1.000–1.009)	0.53 (0.48–0.59)	0.53 (0.48–0.59)	..
XII Other and unspecified malignant neoplasms	0.68 (0.47–0.97)	1.016 (0.996–1.036)	0.67 (0.48–0.95)	0.68 (0.48–0.97)	..
**Children younger than 5 years at time of diagnosis**					
Any cancer	1.13 (1.08–1.18)	1.011 (1.008–1.014)	1.10 (1.05–1.15)	1.11 (1.06–1.16)	10.69
I Leukaemia, myeloproliferative, and myelodysplastic diseases	1.02 (0.95–1.10)	1.012 (1.008–1.017)	1.00 (0.93–1.07)	1.01 (0.94–1.09)	..

Cancer site	Controlled direct effect[a] OR (95% CI)	Natural indirect effect OR (95% CI)	Natural direct effect OR (95% CI)	Marginal total effect OR (95% CI)	Percentage (%) mediated[b]
II Lymphomas and reticuloendothelial neoplasms	2.28 (1.82–2.85)	1.006 (0.999–1.013)	2.27 (1.82–2.83)	2.28 (1.83–2.84)	..
III CNS and miscellaneous intracranial and intraspinal neoplasms	1.16 (1.05–1.28)	1.013 (1.008–1.018)	1.16 (1.05–1.27)	1.17 (1.07–1.29)	8.21
IV Neuroblastoma and other peripheral nervous cell tumours	1.11 (0.97–1.26)	1.010 (1.004–1.016)	1.09 (0.96–1.24)	1.10 (0.97–1.25)	10.51
V Retinoblastoma	1.12 (0.92–1.36)	1.003 (0.996–1.010)	1.11 (0.92–1.35)	1.12 (0.92–1.35)	..
VI Renal tumours	0.89 (0.77–1.04)	1.015 (1.007–1.023)	0.87 (0.75–1.00)	0.88 (0.76–1.02)	..
VII Hepatic tumours	1.78 (1.31–2.41)	1.004 (0.995–1.013)	1.64 (1.22–2.19)	1.64 (1.23–2.20)	..
VIII Malignant bone tumours	1.00 (0.58–1.71)	1.000 (0.987–1.012)	1.00 (0.99–1.01)	1.00 (0.58–1.71)	..
IX Soft tissue and other extraosseous sarcomas	1.29 (1.07–1.56)	1.009 (1.001–1.017)	1.27 (1.06–1.53)	1.28 (1.07–1.54)	4.14
X Germ cell tumours, trophoblastic tumours and neoplasms of gonads	1.58 (1.22–2.04)	1.007 (0.998–1.017)	1.38 (1.09–1.75)	1.39 (1.09–1.77)	..
XI Other malignant epithelial neoplasms and malignant melanomas	0.61 (0.34–1.08)	1.030 (0.990–1.073)	0.62 (0.36–1.07)	0.64 (0.37–1.10)	..
XII Other and unspecified malignant neoplasms	0.56 (0.35–0.91)	1.025 (0.995–1.057)	0.57 (0.36–0.89)	0.58 (0.37–0.91)	..
**Children younger than 1 year at time of diagnosis**					
Any cancer	1.10 (0.99–1.21)	1.025 (1.018–1.033)	1.06 (0.97–1.17)	1.09 (0.99–1.20)	28.15
I Leukaemia, myeloproliferative and myelodysplastic diseases	0.67 (0.53–0.86)	1.043 (1.023–1.063)	0.70 (0.56–0.88)	0.73 (0.59–0.91)	..
II Lymphomas and reticuloendothelial neoplasms	1.13 (0.67–1.88)	1.025 (0.997–1.053)	1.14 (0.70–1.86)	1.17 (0.72–1.90)	..
III CNS and miscellaneous intracranial and intraspinal neoplasms	1.19 (0.94–1.50)	1.037 (1.020–1.053)	1.15 (0.93–1.43)	1.20 (0.97–1.48)	21.56
IV Neuroblastoma and other peripheral nervous cell tumours	1.40 (1.14–1.73)	1.020 (1.009–1.031)	1.36 (1.12–1.66)	1.39 (1.14–1.70)	6.85
V Retinoblastoma	1.00 (0.73–1.39)	1.000 (0.991–1.009)	0.96 (0.70–1.32)	0.96 (0.70–1.32)	..
VI Renal tumours	1.22 (0.85–1.76)	1.024 (1.004–1.045)	1.18 (0.84–1.66)	1.21 (0.86–1.70)	13.90
VII Hepatic tumours	1.60 (0.95–2.72)	1.013 (0.991–1.036)	1.45 (0.88–2.37)	1.47 (0.89–2.40)	..
VIII Malignant bone tumours[†]	..	..	..	..	..
IX Soft tissue and other extraosseous sarcomas	1.42 (0.99–2.05)	1.019 (1.001–1.038)	1.36 (0.96–1.92)	1.38 (0.98–1.95)	6.89
X Germ cell tumours, trophoblastic tumours and neoplasms of gonads	1.07 (0.70–1.63)	1.018 (0.997–1.040)	0.88 (0.60–1.29)	0.90 (0.61–1.32)	..
XI Other malignant epithelial neoplasms and malignant melanomas	1.83 (0.46–7.33)	1.058 (0.976–1.147)	1.83 (0.46–7.33)	1.93 (0.48–7.74)	..
XII Other and unspecified malignant neoplasms	0.45 (0.23–0.91)	1.044 (0.990–1.102)	0.47 (0.25–0.88)	0.49 (0.26–0.91)	..

[a]Estimated for no birth defect. [b]Proportion mediated not calculated when the NDE and NIE were in opposite directions or when the CI for NIE contained the null effect. [†]Less than 5 cases, results not included. Abbreviations: OR; odds ratio; CI, confidence interval; CNS, central nervous system.

Supplementary Table S12. Risk of specific cancers in individuals with selected specific birth defects.

Exposure	Outcome	N cases with BD (%)	OR (95% CI)
**Nervous system defects**			
Spina bifida without anencephaly	Any cancer	25 (0.1%)	4.3 (2.7–6.9)
	III CNS	11 (0.2%)	7.9 (4.1–15)
	III f) CNS unspecified	11 (2.5%)	84 (44–164)
	X Germ cell tumours. trophoblastic tumours and neoplasms of gonads	7 (0.6%)	21 (9.6–47)
	X a) Intracranial and intraspinal germ cell tumours	5 (2.6%)	97 (38–247)
	X b) Malignant extracranial and extragonadal germ cell tumours	<5 (1.6%)	58 (14–239)
Hydrocephaly without spina bifida	Any cancer	32 (0.2%)	10 (6.0–16)
	III CNS	27 (0.6%)	35 (21–59)
	III a) Ependymomas and choroid plexus tumour	<5 (0.6%)	36 (11–118)
	III b) Astrocytoma	<5 (0.2%)	10 (3.2–34)
	III c) Intracranial and intraspinal embryonal tumours	8 (0.9%)	53 (24–115)
	III d) Other gliomas	<5 (0.7%)	46 (14–152)
	III e) CNS Other specified	<5 (0.4%)	26 (8.0–86)
	III f) CNS unspecified	7 (1.6%)	129 (55–304)
	X a) Intracranial and intraspinal germ cell tumours	<5 (1.1%)	56 (13–239)
Microencephaly	Any cancer	<5 (0.0%)	1.7 (0.5–5.6)
**Digestive system**			
Biliary atresia	Any cancer	5 (0.0%)	26 (5.0–134)
	II Lymphomas	<5 (0.1%)	180 (32–1011)
	II b) Non–Hodgkin lymphomas	<5 (0.3%)	394 (62–2501)
**Other**			
Craniosynostosis	Any cancer	5 (0.0%)	1.2 (0.5–3.0)
**Single–gene disorders**			
Neurofibromatosis	Any cancer	36 (0.2%)	38 (19–77)

Exposure	Outcome	N cases with BD (%)	OR (95% CI)
Neurofibromatosis cont.	III CNS	26 (0.5%)	121 (58–252)
	III b) Astrocytoma	11 (0.6%)	129 (54–305)
	III d) Other gliomas	5 (1.1%)	238 (79–718)
	III d2) Mixed and unspecified gliomas	<5 (1.3%)	227 (69–744)
	III e) Other specified	<5 (0.3%)	50 (11–230)
	III f) CNS unspecified	8 (1.8%)	1169 (431–3173)
	IV Neuroblastoma (a)	<5 (0.3%)	59 (16–216)
	IX Soft tissue	6 (0.5%)	109 (39–304)
	IX b2) Nerve sheath tumours	5 (5.8%)	2288 (643–8135)
Tuberous sclerosis	Any cancer	26 (0.1%)	67 (24–193)
	III CNS	24 (0.5%)	269 (93–775)
	III b) Astrocytoma	19 (1.0%)	551 (187–1624)
	III d2) Mixed and unspecified gliomas	<5 (0.6%)	303 (54–1689)
	III f) CNS unspecified	<5 (0.5%)	418 (67–2593)
**Genitourinary system**			
Obstructive genitourinary defects	Any cancer	29 (0.1%)	1.6 (1.1–2.3)
	I Leukaemia	5 (0.1%)	0.9 (0.4–2.3)
	I a) Lymphoid Leukaemia	<5 (0.1%)	0.7 (0.2–2.3)
	I b) Acute myeloid leukaemia	<5 (0.3%)	2.8 (0.7–11)
	II Lymphomas	<5 (0.1%)	1.3 (0.4–4.1)
	III CNS	<5 (0.4%)	0.5 (0.1–1.8)
	III e) CNS Other specified	<5 (0.3%)	2.9 (0.7–12)
	VI Renal tumours	5 (0.5%)	5.4 (2.2–13)
	VI a1) Nephroblastoma	<5 (0.5%)	4.6 (1.7–12)
	IX Soft tissue	<5 (0.3%)	3.6 (1.3–9.8)
	IX a) Rhabdomyosarcomas	<5 (0.7%)	7.3 (2.7–20)
	X Germ cell tumours. trophoblastic tumours, and neoplasms of gonads	<5 (0.3%)	4.2 (1.5–11)
	X b) Malignant extracranial and extragonadal germ cell tumours	<5 (2.3%)	26 (8.2–84)

Exposure	Outcome	N cases with BD (%)	OR (95% CI)
Obstructive genitourinary defects cont.	*X b2) Malignant teratomas of extracranial and extragonadal sites*	<5 (2.5%)	25 (6.1–105)
	XI Other malignant epithelial neoplasms and malignant melanoma	<5 (0.1%)	2.1 (0.5–8.5)
	XII Other and unspecified malignant neoplasms	<5 (1.6%)	18 (4.3–73)
Hypospadias	Any cancer	26 (0.1%)	1.0 (0.7–1.5)
	I Leukaemia	8 (0.2%)	1.2 (0.6–2.4)
	II Lymphomas	<5 (0.1%)	0.8 (0.3–2.6)
	III CNS	<5 (0.1%)	0.5 (0.2–1.5)
	VI Renal tumours	<5 (0.4%)	3.7 (1.4–10)
Hypospadias/ epispadias	Any cancer	38 (0.2%)	1.1 (0.8–1.6)
	I Leukaemia	11 (0.2%)	1.3 (0.7–2.3)
	II Lymphomas	5 (0.2%)	1.1 (0.4–2.6)
	III CNS	6 (0.1%)	0.8 (0.3–1.7)
	VI Renal tumours	5 (0.5%)	3.7 (1.5–9.0)
	XI Other malignant epithelial neoplasms and malignant melanoma	<5 (0.2%)	2.0 (0.6–6.2)
**Eye**			
Congenital cataract	Any cancer	7 (0.0%)	1.4 (0.6–3.1)
	VI Renal tumours	<5 (0.3%)	16 (4.8–51)
**Limb**			
Clubfoot	Any cancer[a]	48 (0.2%)	0.9 (0.7–1.3)
Congenital hip dislocation	Any cancer	34 (0.2%)	1.2 (0.8–1.7)
	VI Renal tumours[a]	<5 (0.4%)	3.2 (1.2–8.5)
**Heart**			
Pulmonary artery anomalies	Any cancer	<5 (0.0%)	1.4 (0.4–4.7)
Pulmonary valve atresia and stenosis	Any cancer	<5 (0.0%)	0.9 (0.2–3.9)
Septal defects	Any cancer	97 (0.5%)	1.4 (1.1–1.7)
	I Leukaemia[a]	22 (0.4%)	1.1 (0.7–1.6)

Exposure	Outcome	N cases with BD (%)	OR (95% CI)
Septal defects cont.	II Lymphomas	18 (0.6%)	2.1 (1.3–3.4)
	II a) Hodgkin lymphoma	8 (0.6%)	2.0 (1.0–4.1)
	II b) Non–Hodgkin lymphomas	7 (0.7%)	2.3 (1.1–4.9)
	II b2) Mature B–cell lymphoma (except Burkitt)	<5 (1.5%)	4.9 (1.8–13)
	II b4) Non–Hodgkin NOS	<5 (0.6%)	1.9 (0.5–7.9)
	II d) Miscellaneous lymphoreticular neoplasms	<5 (1.6%)	3.4 (0.8–14)
	III CNS	15 (0.3%)	0.9 (0.5–1.5)
	III c) Intracranial and intraspinal embryonal tumours	<5 (0.4%)	1.2 (0.5–3.3)
	III d2) Mixed and unspecified gliomas	<5 (1.0%)	3.6 (1.2–11)
	III e) CNS Other specified	<5 (0.5%)	1.4 (0.5–3.8)
	III f) CNS unspecified	<5 (0.5%)	1.3 (0.3–5.3)
	IV Neuroblastoma	6 (0.6%)	1.5 (0.6–3.3)
	IV a) Neuroblastoma and ganglioneuroblastoma	6 (0.6%)	1.5 (0.7–3.3)
	VI Renal tumours	6 (0.6%)	1.6 (0.7–3.7)
	VI a1) Nephroblastoma	6 (0.7%)	1.7 (0.8–3.9)
	VII Hepatic tumours	<5 (1.5%)	4.0 (1.5–11)
	VII a1) Hepatoblastoma	<5 (1.6%)	4.2 (1.3–13)
	VIII Malignant bone tumours	<5 (0.4%)	1.6 (0.6–4.4)
	VIII a) Osteosarcomas	<5 (0.6%)	2.3 (0.7–7.1)
	IX Soft tissue[a]	6 (0.5%)	1.4 (0.6–3.2)
	X Germ cell tumours. trophoblastic tumours and neoplasms of gonads	9 (0.7%)	2.8 (1.4–5.4)
	X b2) Malignant teratomas of extracranial and extragonadal sites	<5 (3.8%)	8.6 (2.7–28)
	X c) Malignant gonadal germ cell tumour	5 (0.6%)	2.4 (1.0–5.9)
	X c1) Malignant gonadal germinomas	<5 (1.8%)	7.6 (1.8–31)
	X c4) Gonadal yolk sac tumour	<5 (1.6%)	4.5 (1.1–18)
	XI Other malignant epithelial neoplasms and malignant melanoma*	6 (0.4%)	1.3 (0.6–2.9)
Ventricular Septal defects	Any cancer	72 (0.4%)	1.2 (1.0–1.6)
	I Leukaemia[a]	16 (0.3%)	0.9 (0.6–1.6)
	II Lymphomas	11 (0.4%)	1.6 (0.9–2.9)

Exposure	Outcome	N cases with BD (%)	OR (95% CI)
Ventricular Septal defects cont.	*II a) Hodgkin lymphoma*	5 (0.4%)	1.6 (0.6–3.8)
	II b) Non–Hodgkin lymphomas	<5 (0.4%)	1.6 (0.6–4.4)
	II b2) Mature B–cell lymphoma (except Burkitt)	<5 (1.1%)	4.5 (1.4–14)
	II d) Miscellaneous lymphoreticular neoplasms	<5 (1.6%)	4.3 (1.1–18)
	III CNS[a]	12 (0.2%)	0.9 (0.5–1.5)
	IV Neuroblastoma[a]	6 (0.6%)	1.8 (0.8–4.1)
	VI Renal tumours	<5 (0.4%)	1.4 (0.5–3.7)
	VI a1) Nephroblastoma	<5 (0.5%)	1.5 (0.5–3.9)
	VII Hepatic tumours	<5 (0.7%)	2.5 (0.6–10)
	VII a1) Hepatoblastoma	<5 (1.1%)	3.5 (0.9–14)
	VIII Malignant bone tumours	<5 (0.2%)	1.0 (0.2–4.0)
	VIII a) Osteosarcomas	<5 (0.4%)	1.9 (0.5–7.5)
	IX Soft tissue[a]	6 (0.5%)	1.8 (0.8–4.0)
	X Germ cell tumours, trophoblastic tumours and neoplasms of gonads	7 (0.6%)	2.6 (1.2–5.6)
	X b2) Malignant teratomas of extracranial and extragonadal sites	<5 (2.5%)	7.3 (1.8–30)
	X c) Malignant gonadal germ cell tumour	<5 (0.5%)	2.4 (0.9–6.3)
	X c1) Malignant gonadal germinomas	<5 (1.8%)	9.1 (2.2–37)
	XI Other malignant epithelial neoplasms and malignant melanoma[a]	5 (0.3%)	1.3 (0.5–3.2)
Atrial Septal defects	Any cancer	35 (0.2%)	1.7 (1.2–2.5)
	I Leukaemia	7 (0.1%)	1.2 (0.6–2.5)
	I a1) Precursor cell leukaemia	7 (0.2%)	1.5 (0.7–3.2)
	II Lymphomas	10 (0.4%)	4.3 (2.3–8.1)
	II a) Hodgkin lymphoma	<5 (0.3%)	3.9 (1.4–10)
	II b) Non–Hodgkin lymphomas	5 (0.5%)	6.0 (2.5–15)
	II b2) Mature B–cell lymphoma (except Burkitt)	<5 (1.1%)	13 (4.2–42)
	II b4) Non–Hodgkin NOS	<5 (0.6%)	7.3 (1.8–30)
	III CNS	5 (0.1%)	1.0 (0.4–2.5)
	III f) CNS unspecified	<5 (0.5%)	4.9 (1.2–20)
	IV Neuroblastoma	<5 (0.2%)	1.6 (0.4–6.6)

Exposure	Outcome	N cases with BD (%)	OR (95% CI)
Atrial Septal defects cont.	*IV a) Neuroblastoma and ganglioneuroblastoma*	<5 (0.2%)	1.7 (0.4–6.7)
	VI a) Nephroblastoma and other non–epithelial renal tumours	<5 (0.3%)	3.0 (1.0–9.4)
	VII Hepatic tumours	<5 (0.7%)	6.8 (1.7–28)
	VIII Malignant bone tumours	<5 (0.2%)	2.8 (0.7–11)
	X Germ cell tumours. trophoblastic tumours and neoplasms of gonads	<5 (0.2%)	3.4 (1.1–11)
	X b2) Malignant teratomas of extracranial and extragonadal sites	<5 (2.5%)	20 (4.7–81)
Patent ductus arteriosus	Any cancer	11 (0.1%)	1.1 (0.6–2.0)
	I Leukaemia	6 (0.1%)	2.1 (0.9–4.8)
	VI Renal tumours	<5 (0.2%)	3.9 (1.0–16)
Transposition of great vessels	Any cancer	8 (0.0%)	1.4 (0.7–3.0)
	III CNS	<5 (0.1%)	3.1 (1.1–8.5)

[a]No sub sites with statistically significant results. Abbreviations: BD, birth defect; OR, odds ratio; CI, confidence interval; CNS, central nervous system; NOS, not otherwise specified.

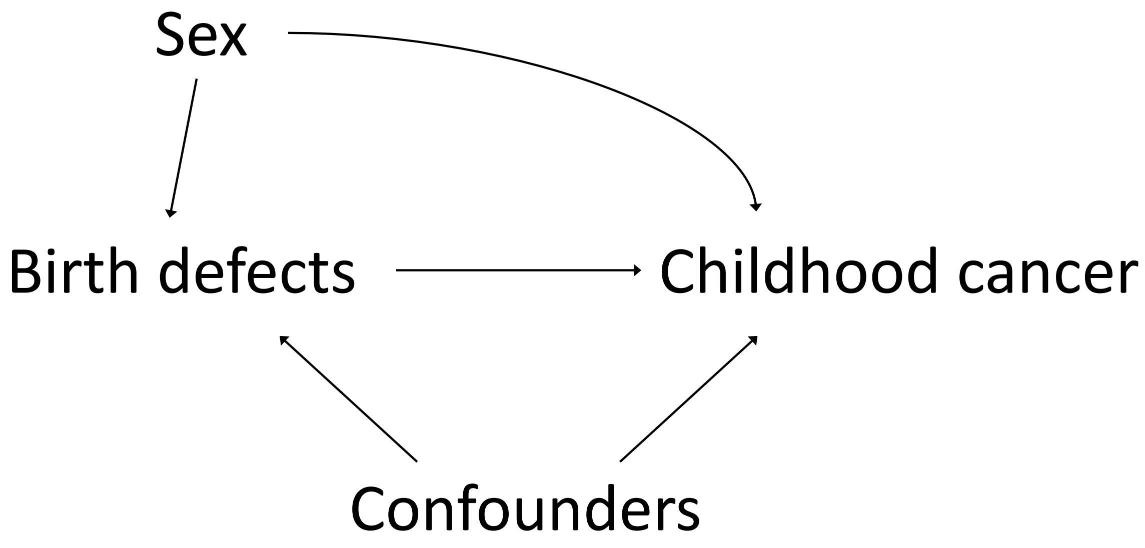

Supplementary Figure S1. A simplified illustration of the assumed causal relationship between sex, birth defects and childhood cancer

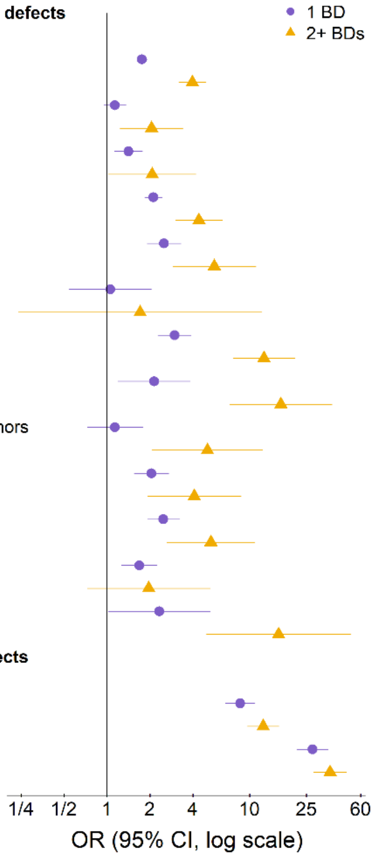

Cancer site	OR (95% CI) 1 BD	OR (95% CI) 2+ BDs	n cases with BD 1 BD	n cases with BD 2+ BDs	P trend
**Non-chromosomal birth defects**					
Any cancer	1.8 (1.6-1.9)	4.0 (3.2-4.9)	869	248	p<0.001
I Leukaemias	1.1 (0.95-1.4)	2.1 (1.2-3.4)	228	129	p=0.005
II Lymphomas	1.4 (1.1-1.8)	2.1 (1.0-4.2)	85	9	p=0.042
III CNS	2.1 (1.8-2.4)	4.4 (3.0-6.5)	220	35	p<0.001
IV Neuroblastoma	2.5 (1.9-3.3)	5.7 (2.9-11)	57	10	p<0.001
V Retinoblastoma	1.1 (0.55-2.1)	1.7 (0.24-12)	9	5	p=0.59
VI Renal tumors	3.0 (2.3-3.9)	13 (7.7-21)	59	21	p<0.001
VII Hepatic tumors	2.2 (1.2-3.8)	17 (7.3-38)	12	7	p<0.001
VIII Malignant bone tumors	1.1 (0.73-1.8)	5.1 (2.1-12)	21	5	p<0.001
IX Soft tissue	2.1 (1.6-2.7)	4.1 (1.9-8.7)	55	8	p<0.001
X Germ cell	2.5 (1.9-3.2)	5.4 (2.6-11)	67	9	p<0.001
XI Other epithelial	1.7 (1.3-2.2)	2.0 (0.73-5.3)	50	5	p=0.18
XII Other neoplasms	2.3 (1.0-5.3)	16 (5.0-51)	6	5	p<0.001
**Chromosomal birth defects**					
Any cancer	8.6 (6.8-11)	12 (9.7-16)	125	134	p<0.001
I Leukaemias	28 (21-36)	37 (28-48)	103	113	p<0.001

Supplementary Figure S2. Risk of cancer according to number of major birth defects (1, 2 or more) in different anatomical subgroups.
Results are presented separately for children with non–chromosomal defects only and those with chromosomal birth defects (and additional non–chromosomal defects). ORs are adjusted for matching variables (birth year and country) and sex. Orthogonal polynomial contrasts were used to test for linear trends. Abbreviations: BD, birth defect; OR, odds ratio; CI, confidence interval; CNS, central nervous system.

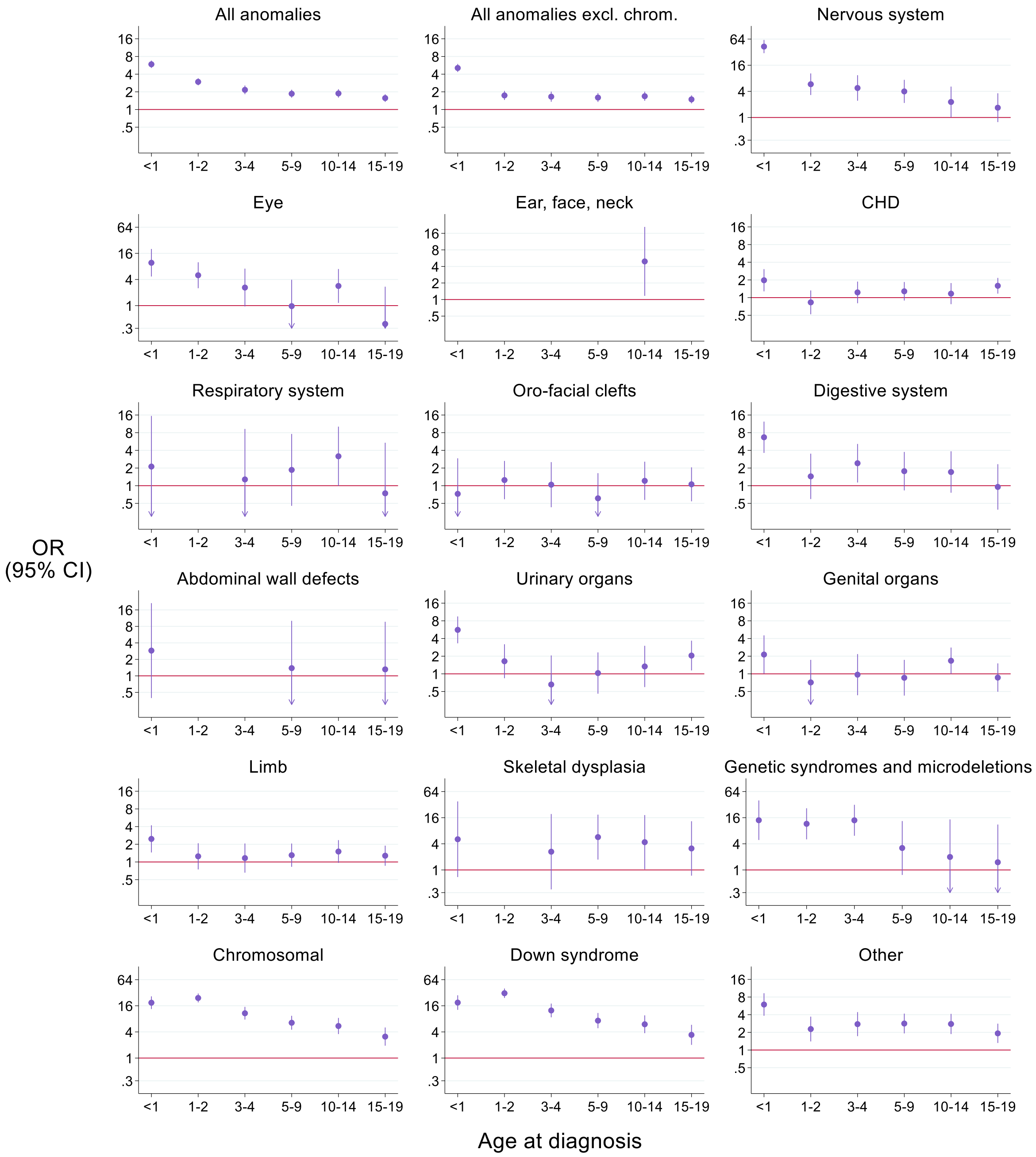

Supplementary Figure S3. Risk of cancer in children with major birth defects, stratified by age at cancer diagnosis.
Some age groups have no co–occurring birth defects and cancer cases. Note that scales differ. ORs are adjusted for matching variables (birth year and country) and sex. Abbreviations: CHD, congenital heart defect; OR, odds ratio; CI, confidence interval.

Supplementary sensitivity analyses – Description of results

When leaving out one country at a time we observed small differences from the results displayed in Figures 1 and 2, and Tables 2-4. Leaving out Finland, resulted in slightly reduced odds ratios (ORs) as expected due to the younger population. The OR for any cancer in children with non-chromosomal birth defects was 1.9 in our main analysis (Figure 1), while leaving out Finland the OR was 1.7. Leaving out Denmark, Norway and Sweden hardly changed the ORs. In Finland, 47% of the children were below the age of five at cancer diagnosis, compared to 35% in Denmark, 38% in Norway, and 34% in Sweden. The only noticeable difference we observed was among children with non-chromosomal birth defects, where the OR of lymphoma was reduced from 1.5 (Figure 2) to 1.1 when excluding Finland and to 1.3 when excluding Sweden, whereas excluding Norway or Denmark increased the OR to 1.7. A total of 26 out of 84 children with both lymphomas and a birth defect had a congenital heart defect. Norway and Denmark had lower numbers of registered heart defects compared to Sweden and Finland, which could explain the reduced association with lymphomas when these countries were included.

Investigating the association between birth defects and cancer stratified by sex when leaving out one country at a time also gave similar results as those found in the main analyses. The ORs of any cancer ranged between 1.8-2.2 among males and 2.6-3.0 among females. Overall, the pattern of greater effect sizes among females than males was observed as in the main results (Tables 2 and 3). For the mediation analyses (Table 4), the overall percentage mediated ranged between 3.4% to 5.7 % when leaving out one country at a time (4.8% in the overall analysis)..

Additional sensitivity analyses including only children born 1990 onwards yielded similar results (slightly higher ORs due to the younger population). Results were similar to those presented in Figures 1-2 and Tables 2-3. The percentage mediated was slightly higher than in Table 4 (from 4.8% to 7.0%). The sex-cancer association (Supplementary Table S2) was slightly reduced (from 1.16 to 1.12 overall), and similarly, the male-to-female sex ratio for birth defects was lower (from 1.30 to 1.23).

Since the germ cell tumour (GCT) group was heterogeneous and types differed in males and females, we did sensitivity analyses for the results presented in Table 2 in which we excluded GCTs. The overall risk of cancer among children with BDs stratified by sex were similar when excluding germ cell tumours. Including GCT: OR_{Males}=2.1 vs. $OR_{Females}$=2.8, excluding GCT: OR_{Males}=2.0 vs. $OR_{Females}$=2.8.
